# Unravelling turbot (*Scophthalmus maximus*) resistance to *Aeromonas salmonicida*: transcriptomic insights from two full-sibling families with divergent susceptibility

**DOI:** 10.3389/fimmu.2024.1522666

**Published:** 2024-12-06

**Authors:** Patricia Pereiro, Ricardo Tur, Miguel García, Antonio Figueras, Beatriz Novoa

**Affiliations:** ^1^ Instituto de Investigaciones Marinas (IIM), Consejo Superior de Investigaciones Científicas (CSIC), Vigo, Spain; ^2^ Nueva Pescanova Biomarine Center, S.L., O Grove, Spain

**Keywords:** turbot families, *Aeromonas salmonicida*, furunculosis, disease resistance, transcriptome sequencing

## Abstract

**Introduction:**

Furunculosis, caused by the gram-negative bacterium *Aeromonas salmonicida* subsp. *salmonicida*, remains a significant threat to turbot (*Scophthalmus maximus*) aquaculture. Identifying genetic backgrounds with enhanced disease resistance is critical for improving aquaculture health management, reducing antibiotic dependency, and mitigating economic losses.

**Methods:**

In this study, five full-sibling turbot families were challenged with *A. salmonicida*, which revealed one family with significantly greater resistance. Transcriptomic analyses (RNA-Seq) were performed on resistant and susceptible families, examining both naïve and 24-h postinfection (hpi) samples from head kidney and liver tissues.

**Results:**

In the absence of infection, differentially expressed genes (DEGs) were identified predominantly in the liver. Following infection, a marked increase in DEGs was observed in the head kidney, with many genes linked to immune functions. Interestingly, the resistant family displayed a more controlled inflammatory response and upregulation of genes related to antigen presentation and T-cell activity in the head kidney at early infection stages, which may have contributed to its increased survival rate. In the liver, transcriptomic differences between the families were associated mainly with cytoskeletal organization, cell cycle regulation, and metabolic processes, including insulin signalling and lipid metabolism, regardless of infection status. Additionally, many DEGs overlapped with previously identified quantitative trait loci (QTLs) associated with resistance to *A. salmonicida*, providing further insights into the genetic basis of disease resistance.

**Discussion:**

This study represents the first RNA-Seq analysis comparing resistant and susceptible turbot families and contributes valuable knowledge for the development of selective breeding programs targeting disease resistance in turbot and other aquaculture species susceptible to *A. salmonicida*.

## 1 Introduction

Turbot (*Scophthalmus maximus*) aquaculture is a significant economic activity in China and several European countries, particularly Spain (1). In 2022, the global production of farmed turbot was estimated at 78,566 tonnes, with China producing approximately 66,000 tonnes and Spain producing approximately 9,000 tonnes (1). According to the FAO, less than 10% of the turbot available in global markets comes from wild capture (2), indicating that the consumption of this species primarily depends on aquaculture production.

However, like other farmed fish species, turbot aquaculture faces persistent challenges related to fish health management. Turbot production is highly susceptible to various bacterial, viral, and parasitic diseases, which can lead to substantial economic losses. The gram-negative bacterium *Aeromonas salmonicida* subsp. *salmonicida* is the aetiologic agent of classical furunculosis, a ubiquitous disease that poses a serious concern, especially for salmonid species (3). However, this bacterium can infect important nonsalmonid farmed fish species, including turbot (4–6). Indeed, a recent bacteriological analysis in different Chinese turbot farms revealed that *Edwardsiella piscicida* and *A. salmonicida* were the most prevalent bacterial pathogens affecting turbot, although the subspecies of *A. salmonicida* was not specified (7).

Even though there are vaccines with certain efficacy against many bacteria, including *A. salmonicida* subsp. *salmonicida*, these can have limitations due to their route of administration (primarily effective through injection), as well as factors such as cost, partial effectiveness, or duration of effectiveness, among others (8). Moreover, the immunocompetence of fish needs to be fully developed for effective vaccination, making it an ineffective strategy in early developmental stages (8). These limitations make antibiotics a widely adopted control strategy during bacterial outbreaks, posing significant threats to human, animal, and environmental health (9, 10). This underscores the urgency of developing new strategies to control bacterial diseases.

Several alternative preventive and control measures have been proposed for the fish aquaculture industry, such as phage therapy, quorum quenching, bacteriocins, the use of probiotics and/or prebiotics, and the use of plant extracts, among others (11). However, comparative immunology provides critical insights into the mechanisms underlying host resistance and susceptibility to pathogens, particularly in aquaculture species where disease outbreaks can have devastating economic and ecological impacts. In this sense, selective breeding programs aimed at producing fish stocks with improved resistance to pathogens have emerged during recent decades as effective complementary strategies (12). Many of these programs rely on family selection, facilitated by the ability to obtain a large number of full siblings for testing purposes (12, 13). Selective breeding programs are based on marker-assisted selection, which uses DNA markers associated with quantitative trait loci (QTLs) that affect a trait of interest, and, more recently, on genome-wide association studies (GWASs), which allow the identification of the complete repertoire of single-nucleotide polymorphisms (SNPs) associated with a trait of interest (14). In turbot, QTL associated to an increased survival to the bacterium *A. salmonicida* (15), the viral hemorrhagic septicemia virus (VHSV) (16) and the ciliate parasite *Philasterides dicentrarchi* (17) have been identified. More recently, GWAS has allowed the identification of the genetic variation contributing to resilience to *P. dicentrarchi* (18). By identifying the genetic and molecular factors that contribute to resistance, breeding strategies can be optimized to develop more resilient fish populations, ultimately improving aquaculture sustainability (19). Analyzing the transcriptomic, proteomic, or metabolic profiles between resistant and susceptible genetic backgrounds provides valuable insights for genetic selective breeding programmes but also aids other selection strategies (e.g., measurement of certain transcripts, proteins, or metabolites in blood samples) and the development of treatments.

During the last few years, microarrays and RNA-Seq analyses have been used to explore the transcriptome differences between fish families with different susceptibilities to infectious diseases. This is the case for Atlantic salmon (*Salmo salar*) families with distinct susceptibilities to infectious pancreatitis necrosis virus (IPNV) (20), the sea louse *Lepeophtheirus salmonis* (21), or the amoeba *Neoparamoeba perurans* (22), for Japanese flounder (*Paralichthys olivaceus*) with different susceptibilities to the bacterium *Edwardsiella tarda* (23), and for rainbow trout (*Oncorhynchus mykiss*) with different resistances to *Flavobacterium psychrophilum* (24) or viral hemorrhagic septicemia virus (VHSV) (25), among others. To the best of our knowledge, transcriptome differences between turbot families with varying susceptibilities to an infectious disease have been analyzed only for VHSV through microarray analysis (26). Previous RNA-Seq studies have allowed us to understand the immune response in the head kidney of turbot infected with *A. salmonicida* subsp*. salmonicida* and even to identify genes and mechanisms that could be relevant for resistance against this bacterium (27, 28). However, comparisons between turbot families with different susceptibilities to *A. salmonicida* remain unexplored. In this study, we assessed the survival of five full-sibling turbot families following challenge with *Aeromonas salmonicida* subsp. *salmonicida*. One family demonstrated significantly greater resistance to the bacteria than the other four families did. The transcriptome profiles of the head kidney and liver from this resistant family and the family with the lowest survival rate, labelled the susceptible family, were analyzed by RNA-Seq under naïve (uninfected) conditions and at 24 hpi. Additionally, we examined the overlap between differentially expressed genes (DEGs) in these families and the seven major QTLs previously associated with *A. salmonicida* resistance in turbot (15).

## 2 Materials and methods

### 2.1 Fish and bacteria

Five full-sibling turbot families (initial body weights of 8–9 g) of the exact same age (identified as 22035, 22037, 22038, 22039 and 22040) were kindly provided by the Pescanova Biomarine Center (Galicia, Spain). The fish were maintained in 500 L fiberglass tanks with a recirculating saline water system (salinity 35 g/L) at 18°C and a 12 L:12 D photoperiod and were fed daily to satiety with a commercial dry diet (GEMMA Diamond 1.5, Skretting). Before the experiments, the fish were acclimatized to laboratory conditions for 2 weeks. For fish injections, the animals were anaesthetized with MS-222 (50 mg/L); before sampling, the fish were sacrificed by a MS-222 overdose (500 mg/L). Fish care and challenge experiments were reviewed and approved by the CSIC National Committee on Bioethics under approval number ES360570202001/21/FUN.01/INM06/BNG01.

The pathogen bacteria *Aeromonas salmonicida* subsp. *salmonicida* was used for the challenges in turbot. Bacteria were cultured on tryptic soy agar (TSA) plates overnight at 22°C. The bacterial suspension was prepared in phosphate-buffered saline (PBS) immediately before injection, and its final concentration was determined by analyzing the number of colony-forming units (CFUs) with 10-fold serial dilutions of the bacterial suspension seeded on TSA plates.

### 2.2 Bacterial challenges to identify turbot families with different degrees of resistance to *A. salmonicida* subsp. *salmonicida*.

Before the challenge, ten individuals from each of the five families were randomly selected, and the length and weight of the turbot were determined with an ichthyometer and a precision scale, respectively. To eliminate the influence of fish size on resistance to bacteria, one-way ANOVA (Tukey’s multiple comparisons test) was used to compare the length and weight of the fish from each family. The significance threshold was set at p < 0.05.

The turbot from each family were distributed into 4 tanks containing 17 turbot each. The individuals from 2 tanks were intraperitoneally (i.p.) injected with 100 µL of an *A. salmonicida* suspension (5.9 × 10^8^ CFU/mL), whereas the individuals from the remaining 2 tanks were i.p. inoculated with the same volume of PBS. For each family, the tanks (two infected and two uninfected) were randomly distributed to avoid the influence of tank position. At 24 hpi, two fish per tank were sampled, and the head kidney, the main hematopoietic tissue in fish, and the liver, the primary metabolic organ, were dissected, resulting in 4 biological replicates per tissue and family from infected and noninfected turbot. The samples were maintained at -80°C until use. The remaining fish in the infected and uninfected tanks (n=15 × 2) were maintained for mortality monitoring over a period of 21 days. Survival data were analyzed with Kaplan–Meier survival curves, and statistically significant differences were determined with a log-rank (Mantel−Cox) test.

### 2.3 RNA isolation, cDNA synthesis, and quantitative PCR (qPCR) for *A. salmonicida* detection and RNA-Seq validation

RNA was isolated with the Maxwell RSC simplyRNA Tissue Kit (Promega) with an automated Maxwell RSC 48 Instrument following the guidelines outlined by the manufacturer. The quantity of RNA was measured with a NanoDrop ND-1000 (NanoDrop Technologies, Inc.). These RNA samples were used not only for Illumina library preparation but also for PCR detection of *A. salmonicida* and validation of the RNA-Seq results by qPCR. For this purpose, cDNA synthesis was conducted with an NZY First-Strand cDNA Synthesis Kit (NZYTech) using 0.5 µg of RNA according to the manufacturer’s instructions.

*A. salmonicida* subsp. *salmonicida* was detected with specific primers designed on the basis of the publication of Balcázar et al. (29) but with some modifications. Three genes that were differentially expressed between the resistant and susceptible families under naïve conditions and in both tissues were used to validate the RNA-Seq results: sentrin-specific protease 2 (*senp2*), E3 ubiquitin-protein ligase HECTD2 (*hectd2*) and toll-like receptor 5a (*tlr5a*). Individual qPCRs were conducted in a final volume of 25 µL, comprised of 12.5 µL of SYBR GREEN PCR Master Mix (Applied Biosystems), 10.5 µL of ultrapure water (Sigma–Aldrich), 0.5 µL of each specific primer (10 µM), and 1 µL of cDNA template. All reactions were performed with technical triplicates with a 7300 Real-Time PCR System thermocycler (Applied Biosystems). The protocol involved an initial denaturation step at 95°C for 10 minutes, followed by 40 cycles of denaturation at 95°C for 15 seconds and hybridization-elongation at 60°C for 1 minute. The relative detection of the bacteria and the expression of the three genes were normalized according to Pfaffl’s method (30), using the eukaryotic elongation factor 1 alpha (*eef1a*) gene as the reference gene. For the validation results, fold-change units were calculated by dividing the normalized expression values of the resistant family by the normalized expression values of the susceptible family. The primer pairs used in this work are listed in **Supplementary Table S1**.

### 2.4 Transcriptome sequencing and RNA-Seq analysis

Three biological replicates per experimental condition (resistant control, resistant infected, susceptible control and susceptible infected) and tissue (head kidney and liver) were selected randomly and used for transcriptome analysis. Double-stranded cDNA libraries were constructed with the TruSeq Stranded mRNA LT Sample Kit (Illumina, San Diego, CA, USA). Paired-end 150 bp (PE150) sequencing was carried out on an Illumina NovaSeq 6000 sequencer. Both library preparation and sequencing were performed at Macrogen, Inc. (Seoul, Republic of Korea). The files containing the raw read sequences were deposited in the Sequence Read Archive (SRA) (http://www.ncbi.nlm.nih.gov/sra) under BioProject accession number PRJNA1178858.

CLC Genomics Workbench v. 22.0 (CLC Bio, Aarhus, Denmark) was used to filter and trim reads and to conduct the RNA-Seq analyses. The raw reads were trimmed to remove adaptor sequences and low-quality reads with a quality score limit of 0.05. RNA-Seq analyses were performed with the turbot genome (31) with the following parameters: length fraction = 0.8, similarity fraction = 0.8, mismatch cost = 2, insertion cost = 3 and deletion cost = 3. The expression values were set as transcripts per million (TPM). Finally, a differential expression analysis test was used to compare gene expression levels and to identify differentially expressed genes (DEGs). Genes with a fold-change (FC) value > |2| and a p value < 0.01 were considered differentially expressed in the different comparisons of interest and retained for further analyses.

### 2.5 *Post hoc* bioinformatic analyses

Principal component analysis (PCA) plots and heatmaps were constructed with the Clustvis web tool (32; https://biit.cs.ut.ee/clustvis/) with the TPM values of the overall transcriptome or the selected transcripts, respectively. For the heatmaps, row and column clustering was conducted with the Euclidean distance and average linkage method. Venn diagrams were drawn with the Venny 2.1.0 tool (http://bioinfogp.cnb.csic.es/tools/venny). Protein−protein interaction networks were constructed with STRING v12.0 software (https://string-db.org) (33).

Gene Ontology (GO) enrichment analyses of the DEGs were performed in OmicsBox v1.3.11 (https://www.biobam.com/omicsbox) with Fisher’s exact test enrichment analysis and a false discovery rate (FDR) ≤ 0.05, and the results were reduced to the most specific terms. Only those biological process terms with a fold enrichment (proportion test/reference) greater than 2 are represented. When more than 30 terms passed this filter, only the 30 most significantly enriched terms were represented. The KEGG mapper tool (34) was used to analyze the main pathways showing differences in gene expression between the resistant and susceptible families.

### 2.6 Integration of DEGs with quantitative trait locus regions associated with *A. salmonicida* subsp. *salmonicida* resistance

The overlap between previously identified major QTL regions associated with *A. salmonicida* subsp. *salmonicida* in turbot (15) and the DEGs between the resistant and susceptible families (both under naïve and infected conditions) was explored. The sequences of the QTL markers were positioned on the turbot genome with the Ensembl Blast tool (https://www.ensembl.org/Multi/Tools/Blast). A conservative narrow window size of 2 Mbp around the QTL-associated markers was considered. The markers and the DEGs positioned around those markers were represented in the turbot genome with MapChart 2.32 (35).

## 3 Results

### 3.1 Evaluation of the survival of different turbot families exposed to *A. salmonicida* subsp. *salmonicida* and evaluation of the bacterial load

To discard body size as a variable influencing the higher or lower survival of the turbot families in response to *A. salmonicida* challenge, the mean length and weight of the turbot from each family were determined (**Supplementary Table S2**). No statistically significant differences were found for either variable among the five full-sibling turbot families.

**Figure 1:**
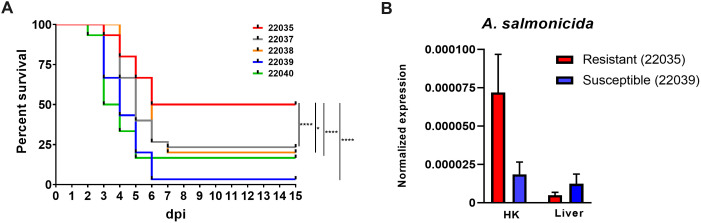
Turbot full-sibling families evaluated for their susceptibility to *A*. *salmonicida* subsp. *salmonicida*. **(A)** Kaplan−Meier survival curves showing the percent survival of the five families analyzed. Statistically significant differences were determined with a log-rank (Mantel−Cox) test and are displayed as **** (p < 0.0001) or * (0.01 < p < 0.05). **(B)** qPCR detection of the bacteria *A*. *salmonicida* subsp. *salmonicida* in the head kidney and liver samples from the most resistant and susceptible families. The detection of the bacteria was normalized to the expression of the *eef1a* gene. The graphs present the means ± SEMs of 4 independent biological replicates. No statistically significant differences were detected between the families.

One of the families, registered as 22035, had a significantly greater survival rate (50%) compared to that of the other four families: 22037 (23.33%), 22038 (20%), 22039 (3.33%) and 22040 (16.67%) (**Figure 1A**). As expected, no mortality events were registered in the mock-challenged tanks. The families 22035 (resistant) and 22039 (susceptible), with the highest and lowest survival rates, respectively, were selected for further analyses.

The bacterial loads in head kidney and liver samples obtained from the resistant and susceptible families at 24 hpi were determined by qPCR (**Figure 1B**). Although no statistically significant differences were detected between the two families, there was a tendency (p =0.09) towards greater detection of *A. salmonicida* in the head kidney samples of the resistant family.

### 3.2 Overall transcriptome comparison between the resistant and susceptible families in the absence and presence of infection

**Figure 2:**
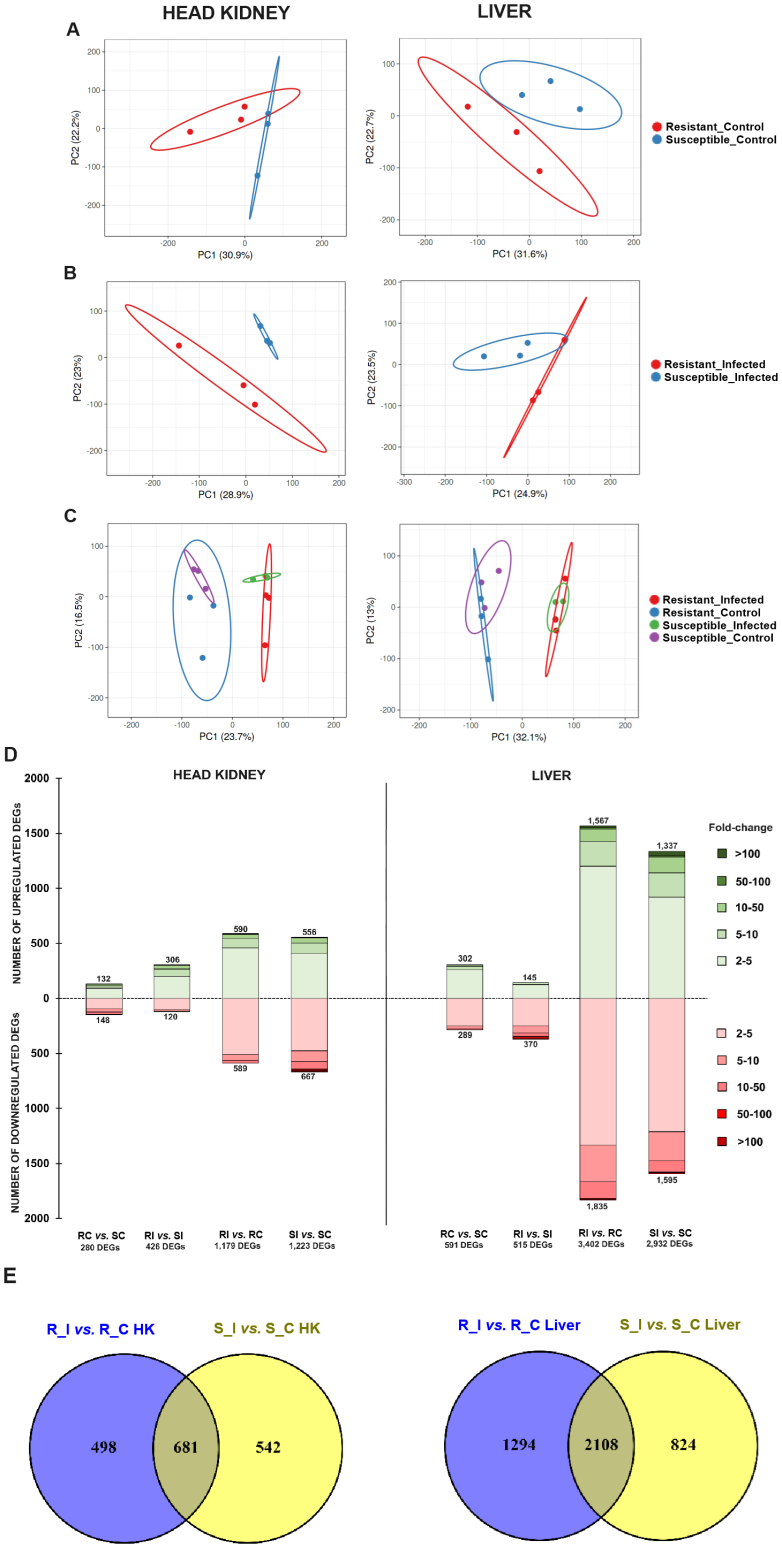
Overall transcriptome comparison of the resistant and susceptible turbot families under naïve and infected conditions and response to bacterial challenge in each family. Principal component analysis (PCA) of head kidney and liver samples from both families under **(A)** naïve (uninfected control), **(B)** *A*. *salmonicida*-infected, and **(C)** all conditions together. **(D)** Stacked column charts representing the number and intensity (in fold-change value) of the DEGs between the different comparisons in head kidney and liver samples. **(E)** Venn diagrams showing the number of shared and exclusive DEGs in response to infection with *A*. *salmonicida* (24 hpi) between both turbot families. R, resistant; S, susceptible; C, control; I, infected.

A summary of the number of raw reads, high-quality reads after trimming, and mapping percentage results per sample is shown in **Supplementary Table S3**. The PCA plots revealed good segregation of the samples from each family under both naïve (**Figure 2A**) and infected (**Figure 2B**) conditions. When all the samples from each tissue were compared in the same plot, as expected, the condition (naïve or infected) accounted for greater transcriptome variation compared to that of the turbot family (resistant or susceptible) (**Figure 2C**).

To identify the differentially expressed genes (DEGs) between different experimental groups, differential expression analyses were conducted on the RNA-Seq results. In the head kidney, the resistant and susceptible families exhibited 280 DEGs (132 up- and 148 downregulated) and 426 DEGs (306 up- and 120 downregulated) under naïve (RC *vs*. SC) and *A. salmonicida*-infected (RI *vs*. SI) conditions, respectively (**Figure 2D**, **Supplementary File S1**). In contrast, in the liver, the number of DEGs was 591 (302 up- and 289 downregulated) and 515 (145 up- and 370 downregulated), respectively (**Figure 2D**, **Supplementary File S2**). Both families presented similar responses to bacterial challenge in terms of the number of DEGs. In the head kidney, the infection modulated 1,179 (590 up- and 589 downregulated) and 1,223 (556 up- and 667 downregulated) genes in the resistant (RI *vs*. RC) and susceptible (SI *vs*. SC) families, respectively; in liver samples, 3,402 (1,567 up- and 1,835 downregulated) and 2,939 (1,337 up- and 1,595 downregulated) genes were modulated by the bacteria in the resistant and susceptible families, respectively (**Figure 2D**, **Supplementary Files S3**, **S4**). Although the number of DEGs in response to the challenge was similar between both turbot families, a substantial number of DEGs were uniquely affected by the bacterium in each family across both tissues. These findings highlight considerable variability in the response, depending on the genetic background of the turbot (**Figure 2E**).

The results were validated by qPCR analysis of three genes differentially regulated between both families and in both tissues: *senp2*, *hectd2*, and *tlr5a*. The expression of these genes under naïve conditions measured by qPCR was highly consistent with the expression results obtained from the transcriptome analysis (**Supplementary Figure S1**).

### 3.3 GO enrichment analyses of the DEGs between the resistant and susceptible families

**Figure 3:**
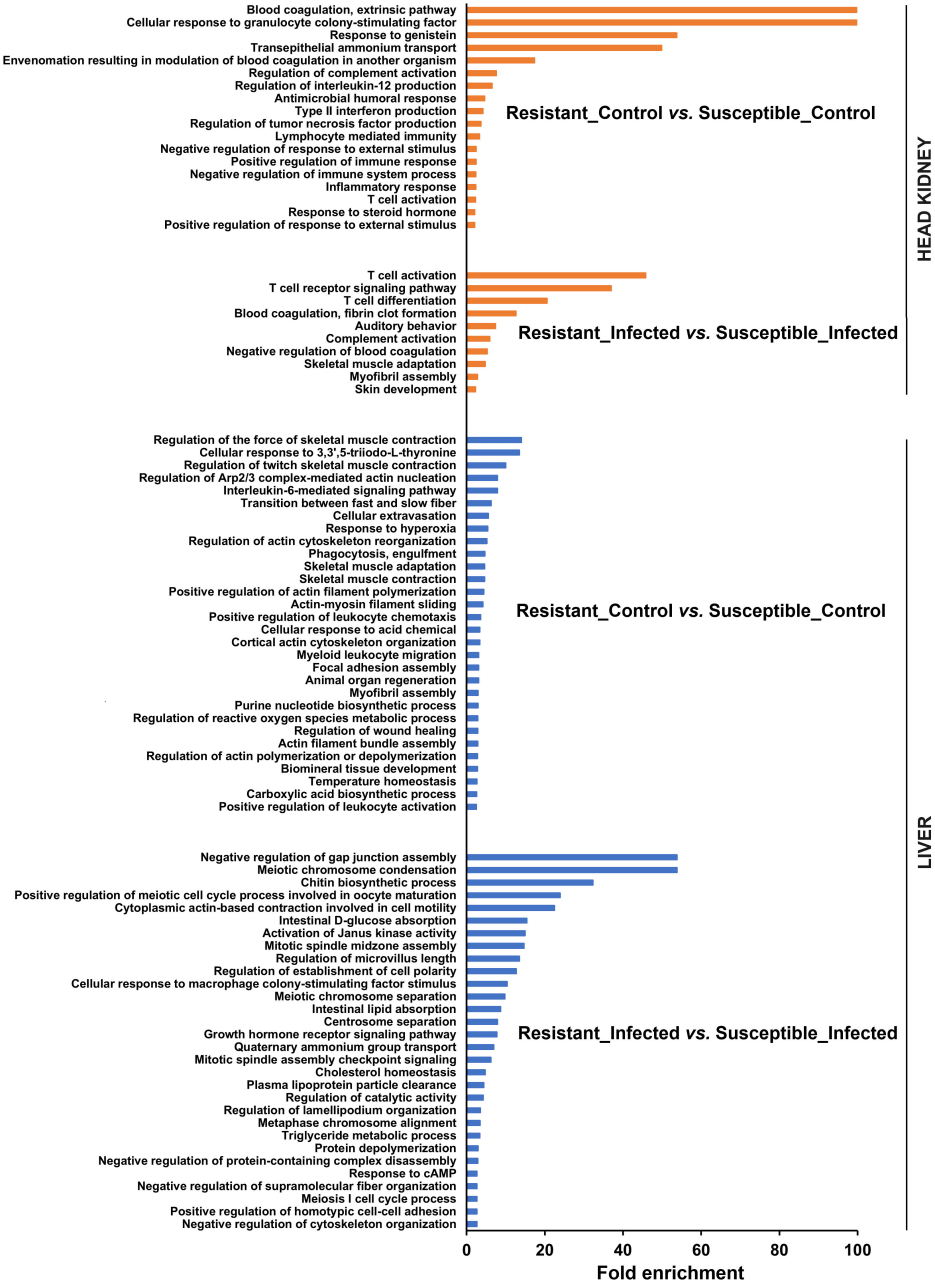
GO enrichment analysis (biological processes) of the DEGs in head kidney and liver samples between both turbot families both in the absence and presence of infection. For the liver, only the 30 most significantly enriched terms are represented.

A comparison of the transcriptomes of both turbot families revealed numerous enriched GO biological process terms (**Figure 3**). In head kidney samples, numerous immune terms were significantly enriched under naïve (or control) conditions, with a strong representation of biological processes related to the complement and blood coagulation pathways, two closely linked processes. Additionally, terms related to antimicrobial activity, cytokine production and activity, and lymphocyte activation were observed. Interestingly, under infected conditions, the terms directly related to immunity were focused primarily on T cells, complement, and blood coagulation.

On the other hand, the number of enriched terms was much greater in the liver (only the 30 most significantly enriched terms are represented), and they were less restricted to immune terms (**Figure 3**). The comparison of both families in the absence of infection revealed an enormous number of terms related to the cytoskeleton. Indeed, most of the enriched immune terms were associated with the cytoskeleton (phagocytosis, extravasation, chemotaxis, and wound healing). In addition, terms related to reactive oxygen species (ROS) and nitric oxide (NO) production and response were also enriched. The comparison of both families at 24 hpi also revealed terms related not only to the cytoskeleton but also to cell division and different metabolic processes, especially those involved in lipid metabolism.

### 3.4 Comparison of head kidney transcriptome profiles between resistant and susceptible families

As determined by the GO enrichment analyses (**Figure 3**), in the head kidney, the transcriptome differences between resistant and susceptible turbot families were characterized mainly by the differential expression of genes directly involved in the immune response (**Supplementary Files S1**, **S2**). This is partially illustrated in **Table 1**, which shows the top 25 genes that were expressed at either higher or lower levels in the resistant family than in the susceptible family, both in the absence and presence of infection.

**Table 1:** Top 25 up- and downregulated DEGs in head kidney samples between the resistant and susceptible turbot families under naïve and infected conditions.

Resistant *vs.* Susceptible (Naïve) - Head kidney			
TOP 25 UP		TOP 25 DOWN	
GENE	FOLD CHANGE	GENE	FOLD CHANGE
Pituitary adenylate cyclase-activating peptide	370.52	Myoglobin	-106.91
P2X purinoceptor	214.57	Lysyl oxidase homolog 4	-105.63
Proteasome subunit beta type-11	109.06	Protein FAM111A	-92.61
Neurofilament medium polypeptide	39.46	Solute carrier family 12 member 3	-32.38
Neurofilament light polypeptide	32.93	Low-density lipoprotein receptor-related protein 2	-24.19
Sentrin-specific protease 2	28.98	Short-chain dehydrogenase/reductase family 42E member 2	-19.77
Insulin gene enhancer protein isl-2a	23.06	Ammonium transporter Rh type B	-18.21
Proteasome subunit beta type-11	21.89	Myosin light chain 3	-17.23
Preprosomatostatin II	16.68	ATP-sensitive inward rectifier potassium channel 1	-15.31
Regulator of G-protein signaling 7a	15.23	Ammonium transporter Rh type C	-14.77
Trafficking regulator of GLUT4 1	14.07	Protocadherin Fat 4	-14.42
Deleted in bladder cancer protein 1	12.64	Prostaglandin D2 receptor 2	-13.86
PHD finger protein 24	11.74	Perforin-1-like	-13.83
Grass carp reovirus (GCRV)-induced gene 2e	11.56	Leucine-rich repeat-containing protein 4B	-13.73
Leucine-rich repeat-containing protein 15	11.28	GTPase IMAP family member 8	-12.44
Dentin matrix acidic phosphoprotein 1	11.17	5-hydroxytryptamine receptor 1D	-12.34
Endonuclease domain-containing 1 protein	10.82	Fibrinogen-like protein 1	-12.26
Collagen, type X, alpha 1a	10.68	Carbonic anhydrase-like	-12.01
Actinodin 2	10.03	Perforin-1-like	-11.91
Carbonic anhydrase 4	9.98	Hepatocyte nuclear factor 4-beta	-11.65
Parvalbumin 8	9.72	Serine/threonine-protein phosphatase with EF-hands 2	-11.25
Deleted in malignant brain tumors 1 protein	9.54	Guanine nucleotide-binding protein subunit alpha-11	-10.64
Regulator of G-protein signaling 7-binding protein	9.47	GTPase IMAP family member 8	-10.12
CD48 antigen	9.32	Haptoglobin	-9.80
MAGUK p55 subfamily member 3	9.27	Solute carrier family 26 member 6	-9.70
Resistant *vs.* Susceptible (Infected) - Head kidney			
TOP 25 UP		TOP 25 DOWN	
GENE	FOLD CHANGE	GENE	FOLD CHANGE
Neurofilament medium polypeptide	342.36	Von Willebrand factor A domain-containing protein 7	-54.22
Transcobalamin-1	172.07	Kinesin-like protein	-18.35
Claudin-15	112.13	Low-density lipoprotein receptor-related protein 2	-12.83
P2Y purinoceptor 3	100.80	Serine/threonine-protein phosphatase with EF-hands 2	-10.40
Ferric-chelate reductase 1	66.47	Potassium voltage-gated channel subfamily C member 1	-9.40
Coagulation factor VII	39.02	Complexin-1	-8.60
Keratin type I cytoskeletal 13	36.64	C1q-related factor	-8.04
Transmembrane protease serine 2	34.88	Synaptonemal complex protein 2	-7.68
Vitamin D3 24-hydroxylase	33.04	G-protein coupled receptor 158	-7.42
Coagulation factor X	31.40	Kinesin-like protein KIF1A	-7.39
Fibrinogen alpha chain	30.11	Contactin-associated protein 1	-6.46
Complement factor H	29.57	Dynein heavy chain 3, axonemal	-6.46
Low choriolytic enzyme	26.79	Von Willebrand factor A domain-containing protein 7	-5.91
Apolipoprotein B-100	24.30	Contactin-associated protein-like 4	-5.87
Myelin protein zero-like protein 2	23.97	Protein FAM111A	-5.52
Basement membrane-specific heparan sulfate proteoglycan core protein	22.15	Sperm-tail PG-rich repeat-containing protein 2	-5.52
Perforin-1-like	20.88	SLIT and NTRK-like protein 4	-5.36
Carboxypeptidase B2	19.60	Beta-2 adrenergic receptor	-5.14
Skin mucus antibacterial l-amino acid oxidase	16.74	T-box transcription factor TBX20	-5.11
Transcobalamin-1	16.16	Myelin regulatory factor-like protein	-4.94
Xin actin-binding repeat-containing protein 2	16.01	CAP-Gly domain-containing linker protein 3	-4.80
Heat shock protein beta-1	15.27	N-terminal EF-hand calcium-binding protein 2	-4.39
Preprosomatostatin II	14.67	Dynamin-3	-4.22
Proteasome subunit beta type-11	14.32	Gamma-aminobutyric acid receptor subunit alpha-3	-4.18
CD48 antigen	14.31	Sodium- and chloride-dependent GABA transporter 3	-4.17

The fold-change values indicate gene expression in the resistant family compared with the susceptible family.

**Figure 4:**
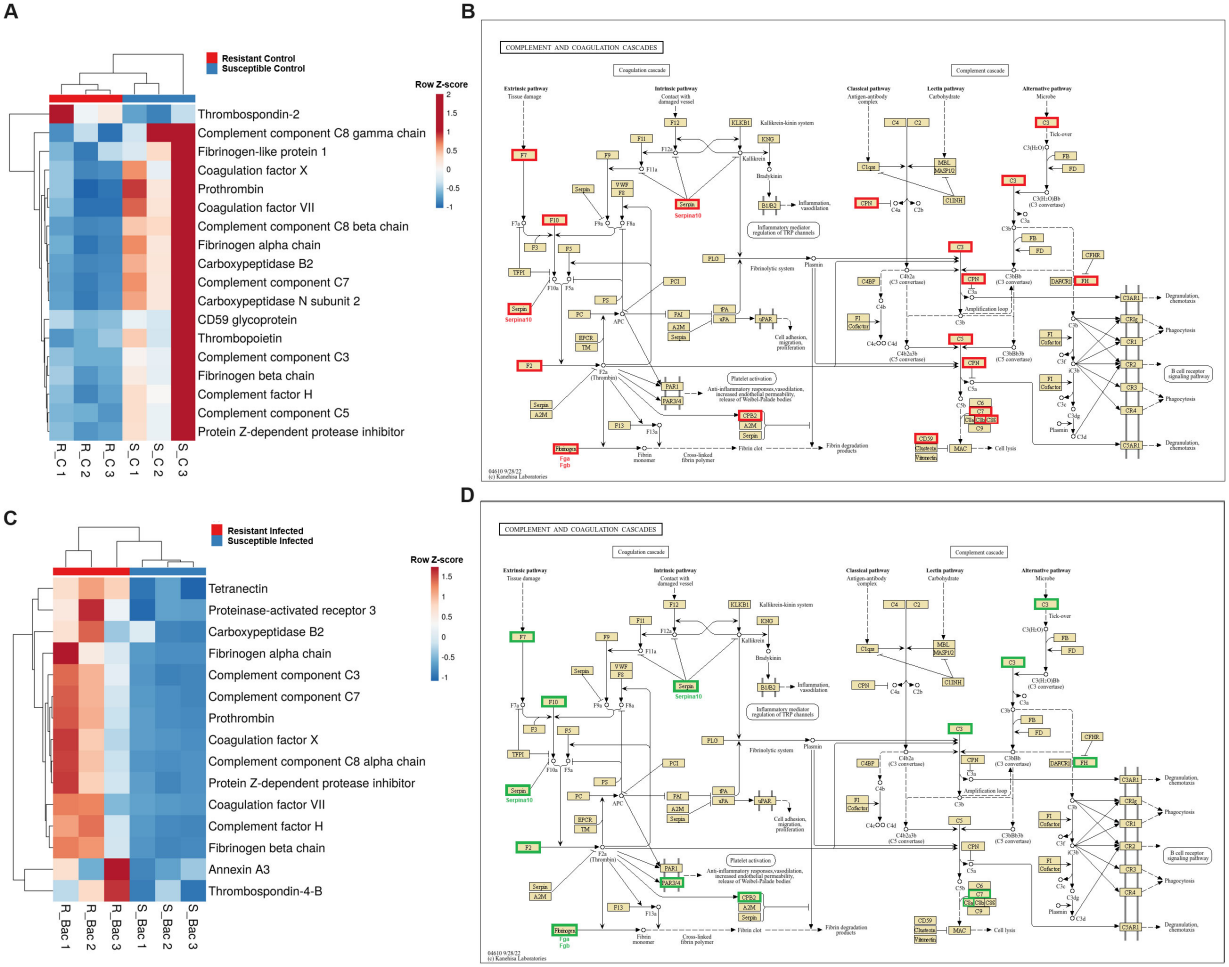
Complement and coagulation genes are differentially expressed in head kidney samples between *A*. *salmonicida*-resistant and *A*. *salmonicida*-susceptible families. **(A)** Heatmap and **(B)** reference KEGG pathways representing the DEGs between both families under naïve conditions and involved in the complement and coagulation cascades. **(C)** Heatmap and **(D)** KEGG reference pathway representing the DEGs between both families at 24 hpi and involved in the “complement and coagulation cascades”. In the KEGG pathways, green indicates higher expression, and red indicates lower expression in the resistant family compared to that in the susceptible family.

**Figure 5:**
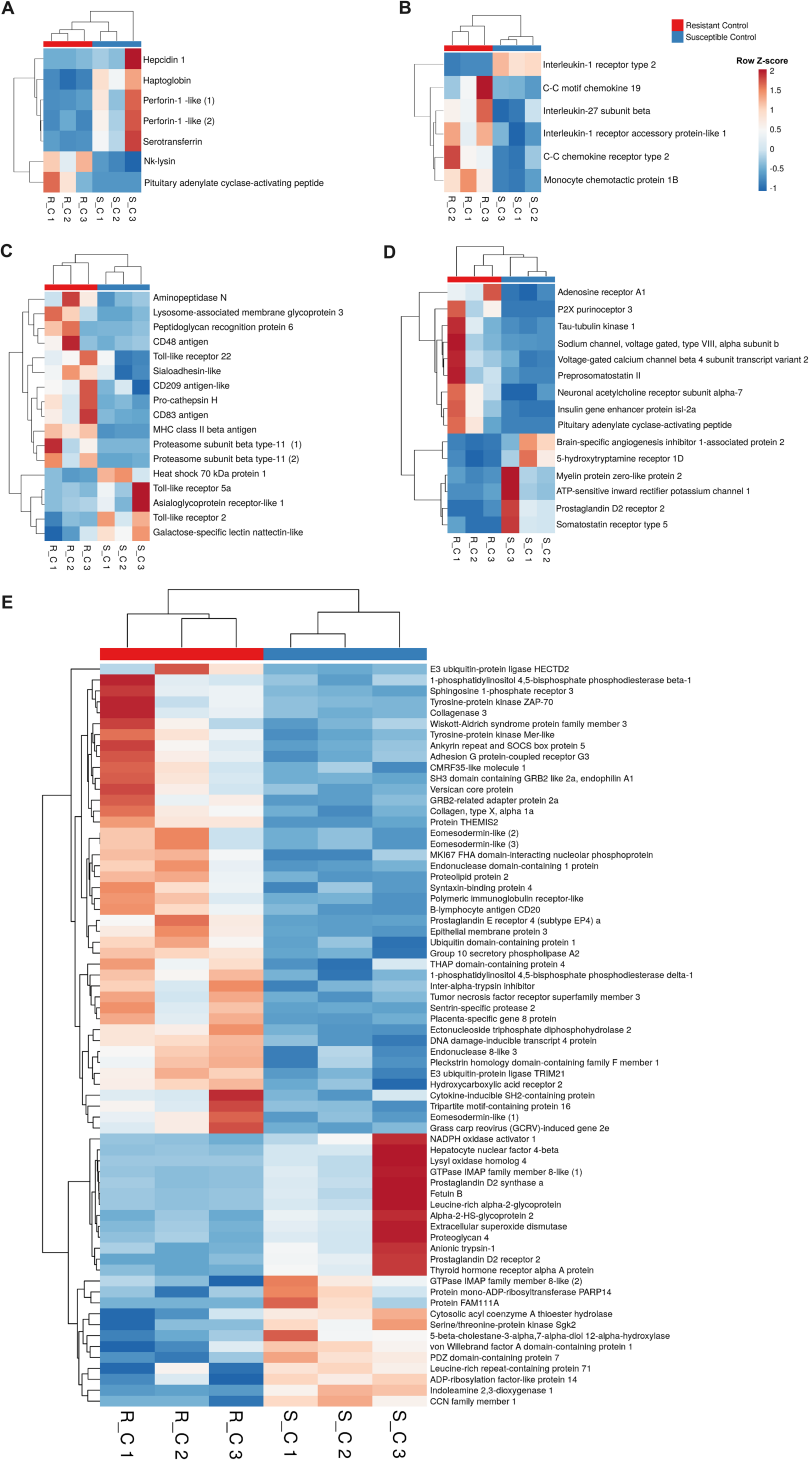
A variety of immune genes are differentially expressed in head kidney samples between the *A*. *salmonicida*-resistant and susceptible families under naïve conditions. In addition to the differences observed in the complement and coagulation pathways in naïve fish, a variety of other immune genes were differentially expressed in the head kidney from both turbot families in the absence of infection. Heatmaps represent the expression (in TPM values) of genes encoding **(A)** antimicrobial and iron-regulatory molecules, **(B)** cytokines and cytokine receptors, **(C)** antigen recognition and presentation-related proteins, **(D)** neuroimmune molecules, and **(E)** other immune proteins.

Since the complement and coagulation pathways exhibited differential expression between the two families, we conducted a detailed analysis of these immune routes. Interestingly, in the absence of infection, the resistant family presented lower expression of several genes involved in these processes than did the susceptible family. These genes included key complement genes such as complement components *c3*, *c5*, *c7*, *c8b* and *c8g*, as well as key coagulation genes such as prothrombin (coagulation factor II), coagulation factor VII, and coagulation factor X, among others (**Figures 4A, B**). Surprisingly, the opposite pattern was observed when the turbot families were compared at 24 hpi, with the complement and coagulation genes showing higher expression in the resistant family (**Figures 4C, D**). Under naïve conditions, many other immune-related genes, such as those encoding antimicrobial peptides and other iron regulatory antibacterial proteins (**Figure 5A**), cytokines and cytokine receptors (**Figure 5B**), pattern recognition receptors (PRRs) and molecules involved in different steps of the antigen presentation process (**Figure 5C**), and neuroimmune genes (**Figure 5D**), among other immune genes (**Figure 5E**), were differentially expressed between the families. The genes encoding the antimicrobial peptides (AMPs) Nk-lysin (*nkl*) and pituitary adenylate cyclase-activating peptide (*pacap)* presented a higher expression level in the resistant family; in contrast, two genes with homology to perforin-1 (*perforin-1-like*) and the gene encoding hepcidin-1 (*hamp1*) were expressed at higher levels in the susceptible family. In addition to its function as an AMP, hepcidin-1 is also involved in the homeostasis of a key bacterial nutrient, iron, as also occurs with serotransferrin and haptoglobin, whose genes were also expressed at higher levels in the susceptible family (**Figure 5A**). Certain cytokines and cytokine receptors were more highly expressed in head kidney samples from the resistant family, with the exception of interleukin-1-receptor type 2 (*il1r2*), a decoy receptor that inhibits the activity of interleukin 1 alpha and beta (**Figure 5B**). Most of the genes that were differentially expressed between both families in head kidney samples under naïve conditions and involved in antigen recognition (peptidoglycan recognition protein 6, sialoadhesin, CD209 antigen, and toll-like receptor 22) and presentation (lysosome and proteasome genes, MHC class II beta antigen, and CD48 or CD83 antigens) presented higher levels of expression in the resistant family (**Figure 5C**). Surprisingly, certain pivotal PRRs for bacterial pathogen-associated molecular patterns (PAMPs), such as toll-like receptor 2 (*tlr2*) and 5a (*tlr5a*), were expressed at lower levels in the resistant family. In addition to *pacap*, other genes involved in both the nervous and immune systems were differentially expressed between families (**Figure 5D**), and most of these genes play a role in regulating the balance between the inflammatory response and tolerance and T-cell activity. Among the other immune genes that were differentially expressed between the two families in the absence of infection in head kidney samples, numerous genes involved in the regulation of the inflammatory/tolerance response and B and T lymphocyte activation and function were observed (**Figure 5E**). For example, the resistant family presented increased transcription of tyrosine-protein kinase ZAP-70 (*zap70*), GRB2-related adapter protein 2a (*grap2a*) and protein THEMIS2 (*themis2*), which control T cells (and B cells in the case of THEMIS2), development, activation, and effector functions, and increased expression of the B-lymphocyte antigen CD20 (*cd20*), a gene encoding a protein with a critical role in B-cell development, activation, antibody production, and immune regulation.

**Figure 6:**
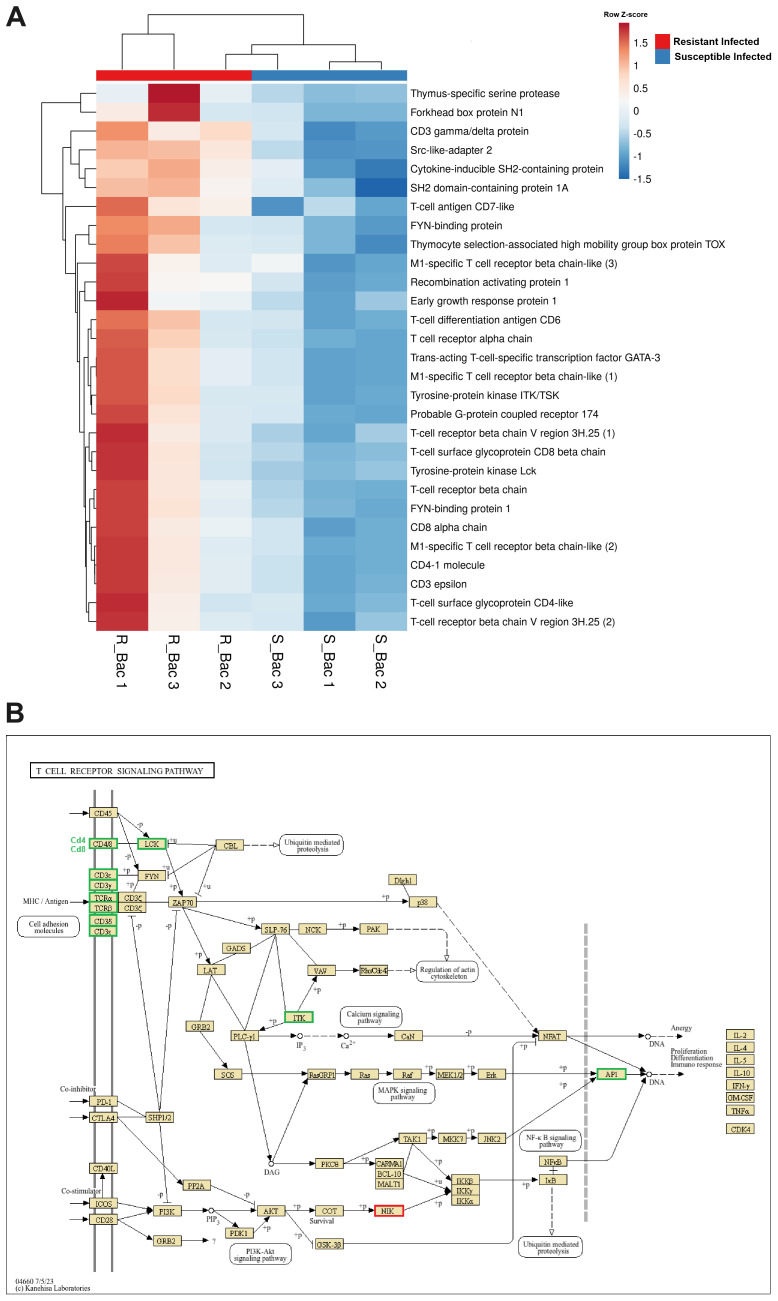
Genes involved in T-cell differentiation, proliferation and activation are expressed at higher levels in the head kidney from the resistant family compared to that from the susceptible family at 24 hpi with *A*. *salmonicida*. **(A)** Heatmap representing the expression (in TPM values) of genes involved in T-cell activity. **(B)** Reference KEGG pathway representing the DEGs in the “T-cell receptor signalling pathway”. In the KEGG pathway, green indicates higher expression, and red indicates lower expression in the resistant family compared to that in the susceptible family.

At 24 hpi with *A. salmonicida*, in addition to complement- and coagulation-related genes, another relevant subset of immune genes differentially expressed between the families was involved in T-cell activity (**Figure 6**). These genes included those encoding various segments of the T-cell receptor (TCR) chains, coreceptor molecules (CD3 epsilon, CD3 gamma/delta, CD4, CD8, CD6, and CD7), and other pivotal genes for T-cell selection, maturation, and activation, such as recombination activating protein 1 (*rag1*), thymus-specific serine protease (*prss16*), thymocyte selection-associated high mobility group box protein TOX (*tox*), tyrosine-protein kinase Lck (*lck*), tyrosine-protein kinase ITK/TSK (*itk*), and trans-acting T-cell-specific transcription factor GATA-3 (*gata3*), among others.

### 3.5 Comparison of the liver transcriptome profiles between the resistant and susceptible families

In the liver, the transcriptome differences between resistant and susceptible turbot families under naïve and infected conditions were characterized mainly by the differential expression of a multitude of genes involved in cytoskeleton organization and related processes, although immune terms were also observed (**Figure 3**, **Supplementary Files S3**, **S4**). This is also reflected in **Table 2**, which shows the top 25 DEGs that were expressed either at higher or lower levels in the liver samples from the resistant family compared to those in the susceptible family.

**Table 2:** Top 25 up- and downregulated DEGs in liver samples between the resistant and susceptible turbot families under naïve and infected conditions.

Resistant *vs.* Susceptible (Naïve) - Liver			
TOP 25 UP		TOP 25 DOWN	
GENE	FOLD CHANGE	GENE	FOLD CHANGE
Neurofilament medium polypeptide	215.36	Disabled -like 2	-67.59
Protein FAM151A	67.67	Zonadhesin	-32.62
Sentrin-specific protease 2	51.56	Synaptopodin 2-like protein	-23.26
Arachidonate 12-lipoxygenase, 12R-type	21.69	FRAS1-related extracellular matrix protein 1	-22.15
Tektin-4	20.51	Pkinase multi-domain protein	-19.00
Zona pellucida sperm-binding protein 3	16.23	Potassium voltage-gated channel subfamily H member 6	-17.42
Zinc transporter 2-like	15.43	Hepcidin 1 precursor	-15.04
Chemokine receptor 5	12.48	Immunoglobulin superfamily DCC subclass member 3	-14.70
Docking protein 3	11.73	Sperm acrosome membrane-associated protein 4	-13.87
Endonuclease domain-containing 1 protein	10.96	GMP reductase 1	-13.49
DNA damage-inducible transcript 4 protein	10.72	Fos-related antigen 1a	-13.11
Spondin-2b	9.77	U11/U12 small nuclear ribonucleoprotein 25 kDa protein	-12.33
Double-stranded RNA-specific editase 1	9.41	Fer-1-like protein 4	-11.60
E3 ubiquitin-protein ligase HECTD2	9.30	Adenine nucleotide translocase lysine N-methyltransferase	-11.44
Prostaglandin E receptor 4 (subtype EP4) a	8.63	Zona pellucida sperm-binding protein 4	-10.11
Ribosomal protein S6 kinase-like 1	8.55	ATP-binding cassette sub-family A member 1	-9.21
B-lymphocyte antigen CD20	7.72	Synaptonemal complex protein 2	-8.67
Acetylcholine receptor subunit beta	7.58	Early growth response protein 1	-8.38
Membrane-spanning 4-domains subfamily A member 4A	7.51	GTP-binding protein RAD	-8.21
B-cell receptor CD22	7.33	Telomere repeats-binding bouquet formation protein 1	-8.09
N-acetyllactosaminide beta-1,3-N-acetylglucosaminyltransferase 3	7.13	Transcription factor Sox-18B	-7.82
C2 domain protein	6.87	Myocardin	-7.82
Src-like-adapter 2	6.19	Tonsoku-like protein	-6.80
GRB2-related adapter protein 2a	5.87	Myosin-10	-6.62
Protein THEMIS2	5.85	DNA polymerase epsilon catalytic subunit A	-6.61
Resistant *vs.* Susceptible (Infected) - Liver			
TOP 25 UP		TOP 25 DOWN	
GENE	FOLD CHANGE	GENE	FOLD CHANGE
Pappalysin-2	26.99	Ladderlectin-like	-9958.07
Protein MRVI1-like	25.83	Angiotensin-converting enzyme 2	-3671.10
Plastin-1	19.49	Aminopeptidase N	-1852.92
Trans-1,2-dihydrobenzene-1,2-diol dehydrogenase	9.95	Glyco hydro 18 multi-domain protein	-1622.38
Sentrin-specific protease 2	9.18	Acidic mammalian chitinase-like	-961.02
Tumor protein p53-inducible nuclear protein 1	7.87	Type-4 ice-structuring protein LS-12	-596.29
E3 ubiquitin-protein ligase HECTD2	7.83	Alkaline phosphatase	-458.33
Wiskott-Aldrich syndrome protein family member 3	7.27	Enteropeptidase-like	-328.58
XK-related protein	7.17	Alpha-tectorin	-327.49
SLIT and NTRK-like protein 3	6.20	Oligopeptide transporter	-304.87
Prolactin receptor	6.01	Alkaline phosphatase, tissue-nonspecific isozyme	-289.20
Calcium/calmodulin-dependent protein kinase II inhibitor 2	5.94	Complement C1q-like protein 4	-211.79
DNA damage-inducible transcript 4 protein	5.89	Glutaredoxin domain-containing cysteine-rich protein 1	-206.59
Prostaglandin G/H synthase 1	5.61	Cytochrome c oxidase subunit NDUFA4	-199.22
Transcription factor COE1	5.56	Relaxin-3 receptor 1-like	-165.07
Neuropilin-1a	5.55	Cytoglobin-1	-154.77
Neurofilament light polypeptide	5.10	G-protein coupled receptor 128	-151.75
Protein-tyrosine kinase 2-beta	4.85	Chitin synthase 1	-148.35
Glutathione-specific gamma-glutamylcyclotransferase 1	4.85	Meprin A subunit beta	-138.86
Serpin H1	4.75	Carboxypeptidase O	-138.59
Neuropilin-1a	4.67	Sodium-dependent neutral amino acid transporter B(0)AT1	-137.75
Protein very KIND	4.63	Natterin-3-like	-116.57
Dystrobrevin alpha	4.56	Chitin synthase 1	-104.87
Serine/threonine-protein kinase ULK1	4.52	Lactase-phlorizin hydrolase	-53.62
Transient receptor potential cation channel subfamily M member 3	4.41	Leucine-rich repeat-containing protein 19	-45.79

The fold-change values indicate gene expression in the resistant family compared with the susceptible family.

**Figure 7:**
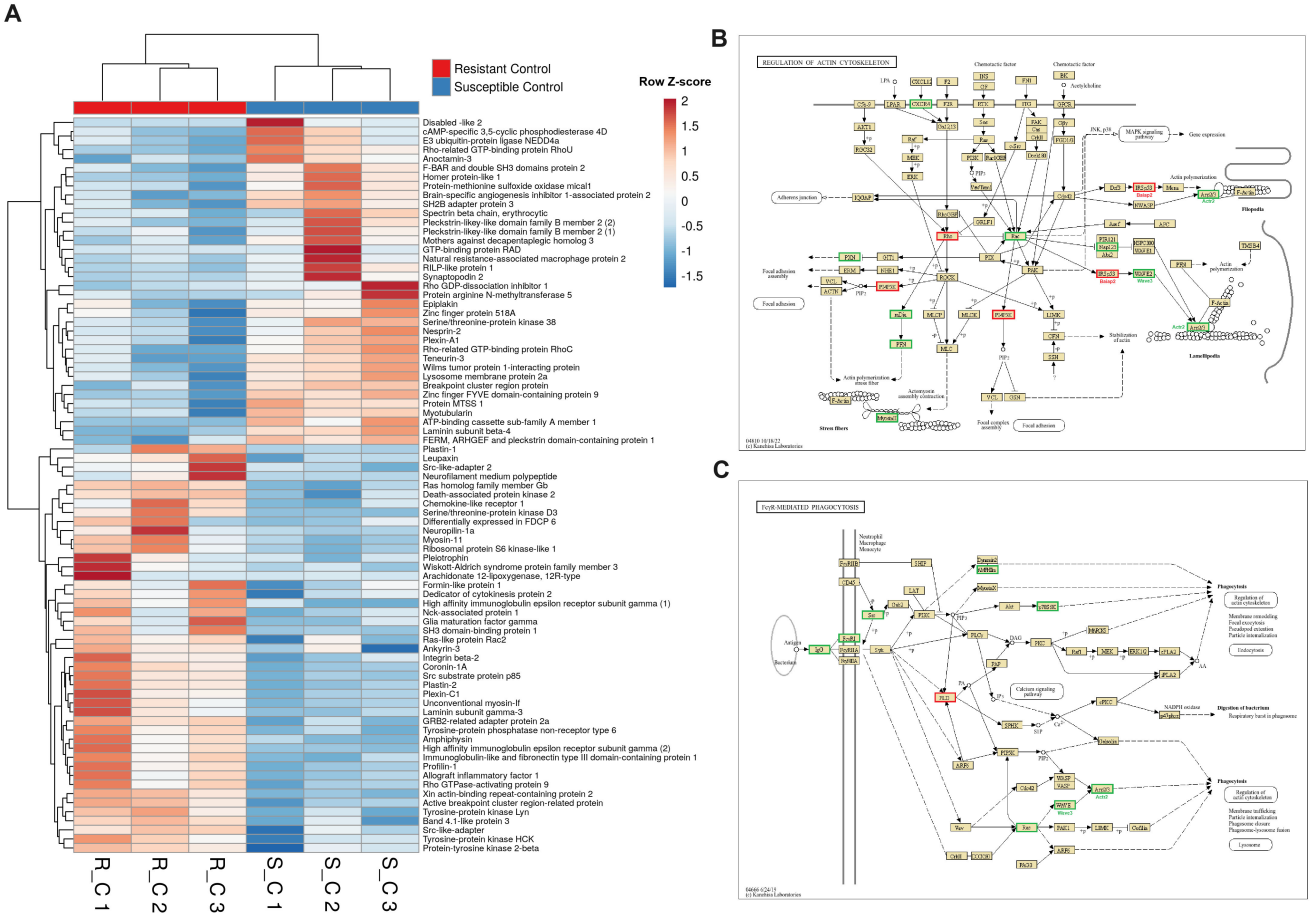
Genes involved in cytoskeleton organization are significantly different in liver samples from both turbot families under naïve conditions. **(A)** Heatmap representing the expression (in TPM values) of genes involved in cytoskeleton organization (including phagocytosis- and chemotaxis-related genes). **(B, C)** Reference KEGG pathways “Regulation of the actin cytoskeleton” and “FcγR-mediated phagocytosis” representing the DEGs between both turbot families. In the KEGG pathways, green indicates higher expression, and red indicates lower expression in the resistant family compared to that in the susceptible family.

**Figure 8:**
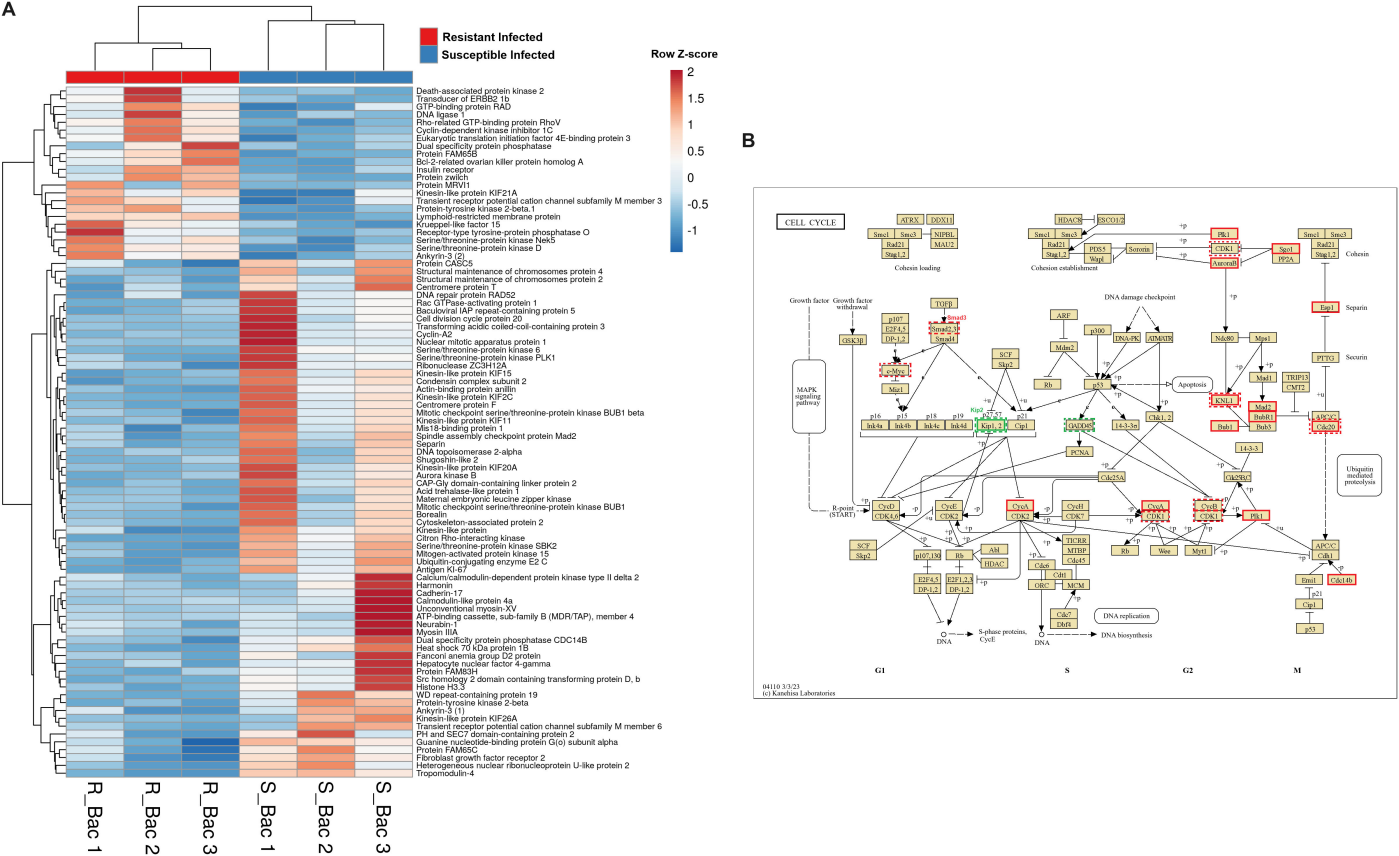
Genes involved in different steps of the cell cycle, such as chromatin condensation, DNA replication, mitotic spindle formation, and cytokinesis, are highly differentially expressed in liver samples from both turbot families at 24 hpi. **(A)** Heatmap representing the expression (in TPM values) of genes involved in the cell cycle. **(B)** Reference KEGG pathway “Cell cycle”, which represents the DEGs between both families under naïve and infected conditions. In the KEGG pathways, green indicates higher expression, and red indicates lower expression in the resistant family compared to that in the susceptible family. The dashed line represents the DEGs between both families under naïve conditions, whereas the solid line represents the DEGs between both families at 24 hpi with *A*. *salmonicida*.

A heatmap including those DEGs classified with GO terms related to the cytoskeleton (muscle contraction, phagocytosis, chemotaxis or actin polymerization-related terms, among others) revealed that approximately half of the genes were more highly expressed in the liver from the resistant family and the other half were more highly expressed in the susceptible family under naïve conditions (**Figure 7A**). Some of the DEGs with higher expression in the resistant family are directly involved in the immune response and are expressed mainly in leukocytes, such as plastin-2 (also known as L-plastin or lymphocyte cytosolic protein 1; *lcp1)*, leupaxin (*lpxn*), src-like adaptor protein 2 (*slap2*), tyrosine-protein kinase Lyn (*lyn*) and two genes annotated as high-affinity immunoglobulin epsilon receptor subunit gamma (*fcer1g*), among others. Broadly speaking, most of the DEGs between both families in liver samples and included in the KEGG pathway “Regulation of the actin cytoskeleton” were more highly expressed in the resistant family (**Figure 7B**). The same was observed for another of the enriched cytoskeleton-related KEGG pathways, “FcγR-mediated phagocytosis”, which is pivotal for the clearance of pathogens (**Figure 7C**). At 24 hpi, some of these differences in the expression of cytoskeleton-related genes were maintained, but in agreement with the enrichment analysis (**Figure 3**), a strong representation of cytoskeleton-related DEGs directly involved in different steps of the cell cycle was observed (**Supplementary Figure S2**). A heatmap representing the expression of these DEGs after infection in both families revealed that most of the genes that play a significant role in cell cycle progression were downregulated in the resistant family compared with the susceptible family (**Figure 8A**). The representation of the DEGs between both turbot families belonging to the KEGG pathway “Cell cycle” also reflected this fact (**Figure 8B**). Indeed, several key cell cycle genes were already differentially expressed in the liver from the naïve individuals, such as cyclin-dependent kinase 1 (*cdk1*), kinetochore scaffold 1 (also known as CASC5; *knl1*), cell division cycle protein 20 (*cdc20*) and cyclin B (*ccnb1*), which showed a lower expression in the resistant family, and the cyclin dependent kinase inhibitor 1C (*cdkn1c*, *kip2* or *p57*) and the growth arrest and DNA damage-inducible protein GADD45 beta (*gadd45b*), which showed a higher expression in the resistant family. The lower expression of genes favoring cycle progression and the higher expression of two key genes involved in cell cycle arrest suggests that liver cells from the resistant family had reduced mitotic activity. After infection, most of those genes were also found to be differentially expressed between both families, with the exception of *cdk1*, *ccnb1* and *gadd45b*, but new genes involved in this process presented lower transcription levels in the resistant family: polo-like kinase 1 (*plk1*), aurora kinase B (*aurkb*), shugoshin-like 2 (*sgo2*), separin (*espl1*), spindle assembly checkpoint protein Mad2 (*mad2*), mitotic checkpoint serine/threonine-protein kinase BUB1 beta (*bubr1*), mitotic checkpoint serine/threonine-protein kinase BUB1 (*bub1*), dual specificity protein phosphatase CDC14B (*cdc14b*) and cyclin A2 (*ccna2*), among others. Motor proteins, which play a fundamental role during different steps of cell division and in the transport of different cargoes (e.g., organelles, vesicles, protein complexes, etc.), also generally presented lower transcription levels in the liver of the turbot-resistant family under both naïve and infected conditions, especially genes encoding kinesins and myosins (**Supplementary Figure S3**).

**Figure 9:**
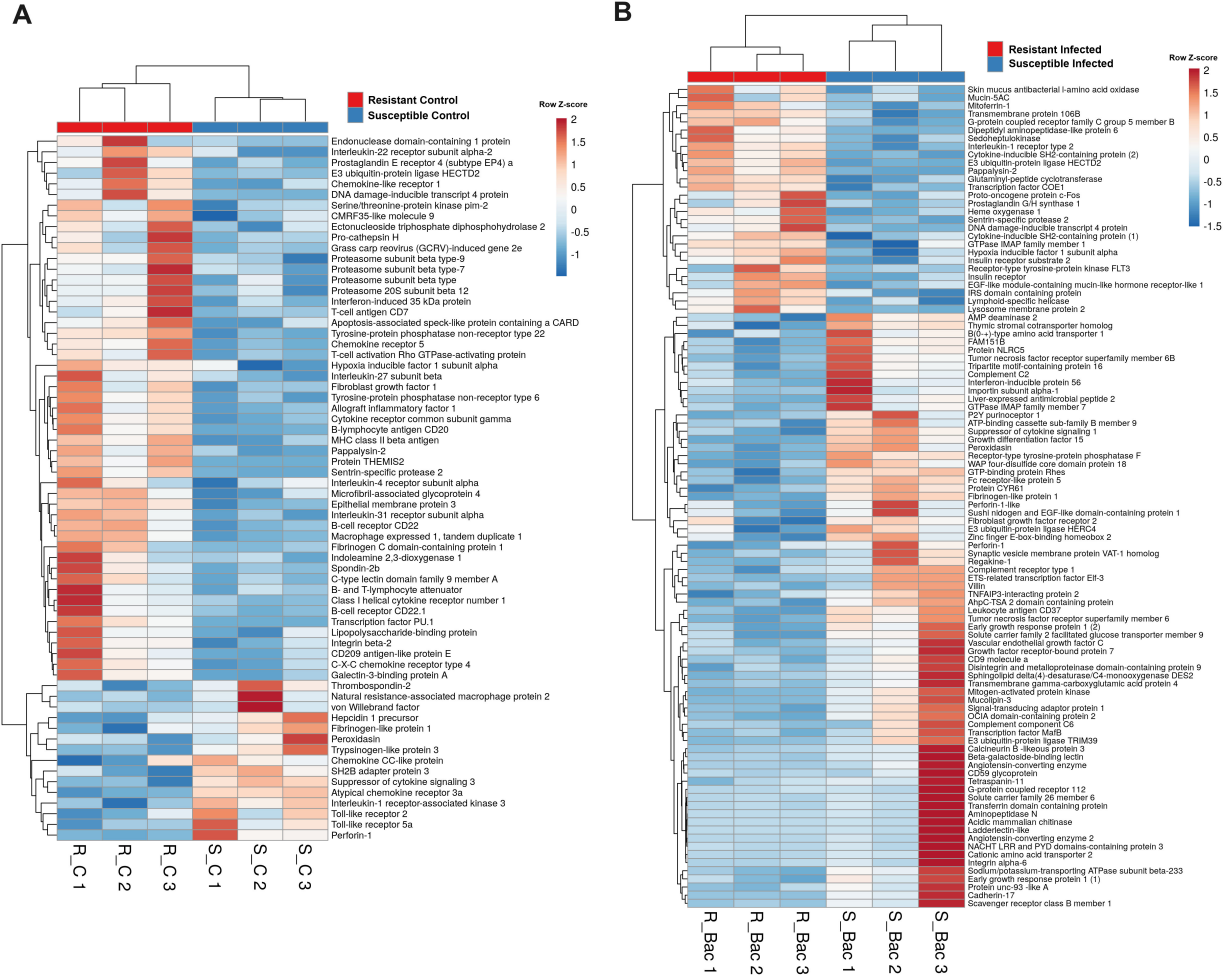
Heatmap representing the main immune genes differentially expressed in the liver samples from resistant and susceptible turbot families **(A)** under naïve conditions and **(B)** at 24 hpi with *A*. *salmonicida*.

However, in addition to those genes linked to the cytoskeleton and cell division, numerous genes involved in the immune response were also differentially expressed in the liver between the turbot families both in the absence (**Figure 9A**) and presence (**Figure 9B**) of infection. In agreement with the findings in the head kidney samples, lower expression of the genes encoding the antimicrobial proteins perforin-1-like, hepcidin-1 (*hamp1*) and liver-expressed antimicrobial peptide 2 (*leap2*) was detected in the resistant family under both naïve (**Figure 9A**) and/or infected (**Figure 9B**) conditions. Additionally, according to the expression observed in the head kidney, *tlr2* and *tlr5a* were expressed at lower levels in the turbot of the resistant family under naïve conditions (**Figure 9A**). Interleukin-1 receptor-associated kinase 3 (*irak3*), a negative regulator of TLR signalling, was also expressed at lower levels in the resistant family. Some immune genes associated with the resistant family in liver samples under naïve conditions were also associated with antigen recognition (lipopolysaccharide-binding protein (*lbp*), C-type lectin domain family 9 member A (*clec9a*), and CD209 antigen-like protein E (*cd209e*)) and presentation (pro-cathepsin H (*ctsh*), macrophage expressed 1, tandem duplicate 1 (*mpeg1.1*), four genes encoding different proteasome subunits and the MHC class II beta antigen) (**Figure 9A**). Additionally, in uninfected fish, several cytokines and cytokine receptors were expressed at higher levels in the resistant family (interleukin-27 subunit beta (*il27b*), interleukin-4 receptor subunit alpha (*il4r*), interleukin-22 receptor subunit alpha 2 (*il22ra2*), interleukin-31 receptor subunit alpha (*il31ra*), chemokine-like receptor 1 (*cmklr1*), C-X-C chemokine receptor type 4 (*cxcr4*), chemokine receptor 5 (*ccr5*), cytokine receptor common subunit gamma (*il2rg*) and a gene annotated as class I helical cytokine receptor number 1), with the exception of two genes annotated as chemokine CC-like protein and atypical chemokine receptor 3a (*ackr3*) (**Figure 9A**). The gene suppressor of cytokine signalling (*socs3*), which is involved in the negative regulation of cytokines, was also expressed at lower levels in the resistant family. Genes with a role in the regulation of the inflammatory/tolerance response and B and T lymphocyte activation and function also showed, in general, higher expression in the resistant family. This was the case for certain lymphocytic antigens (*cd7*, *cd20* and two genes annotated as *cd22*), T-cell activation Rho GTPase-activating protein (*tagap*), B- and T-lymphocyte attenuator (*btla*), transcription factor PU.1 (*spi1*), protein THEMIS2 (*themis2*), hypoxia inducible factor 1 subunit alpha (*hif1a*), tyrosine−protein phosphatase nonreceptor type 22 (*ptpn22*), apoptosis-associated speck-like protein containing a CARD (*pycard*), allograft inflammatory factor 1 (*aif1*), endonuclease domain-containing 1 protein (*endod1*), prostaglandin E receptor 4 (subtype EP4) a (*ptger4a*) and indoleamine-2,3-dioxygenase 1 (*ido1*), among others (**Figure 9A**).

Differences in immune genes were also observed in the liver samples from *A. salmonicida*-infected turbot. Although a few genes remained differentially expressed between both families after challenge, a new repertoire of DEGs was observed (**Figure 9B**). Among these genes, certain genes involved in the complement cascade were expressed at lower levels in the resistant family (*c2*, *c6*, *cr1* or *cd59*), in contrast to what was found in head kidney samples after bacterial infection. A relevant antibacterial gene, skin mucus antibacterial L-amino acid oxidase (*il4i1*), showed an increased transcription level in the liver of turbot of the resistant family (**Figure 9B**). Interestingly, among the few immune-related genes whose expression was increased in the resistant family after infection, an intriguing representation of genes involved in insulin and insulin-like growth factor (IGF) signalling was identified. These genes included insulin receptor (*insr*), insulin receptor substrate 2 (*irs2*), a gene annotated as an IRS domain-containing protein, and pappalysin-2 (*pappa2*) (**Figure 9B**). Notably, *pappa2* was already expressed at higher levels in naïve turbot of the resistant family (**Figure 9A**). A lower expression of another pivotal gene involved in metabolism and immune response control, growth differentiation factor 15 (*gdf15*), which is also a marker of sepsis severity, was observed in the resistant family. As shown by the GO enrichment analysis (**Figure 3**), another relevant metabolic process that seemed to be differentially regulated in the liver after infection was lipid metabolism. Indeed, a heatmap constructed with the genes included in those lipid metabolism-related GO terms reflected this difference (**Supplementary Figure S4**). In general, genes encoding lipid transporter proteins were expressed at lower levels in the resistant family than in the susceptible family.

### 3.6 Shared DEGs in the head kidney and liver that are potentially associated with resistance to *A. salmonicida*

**Figure 10:**
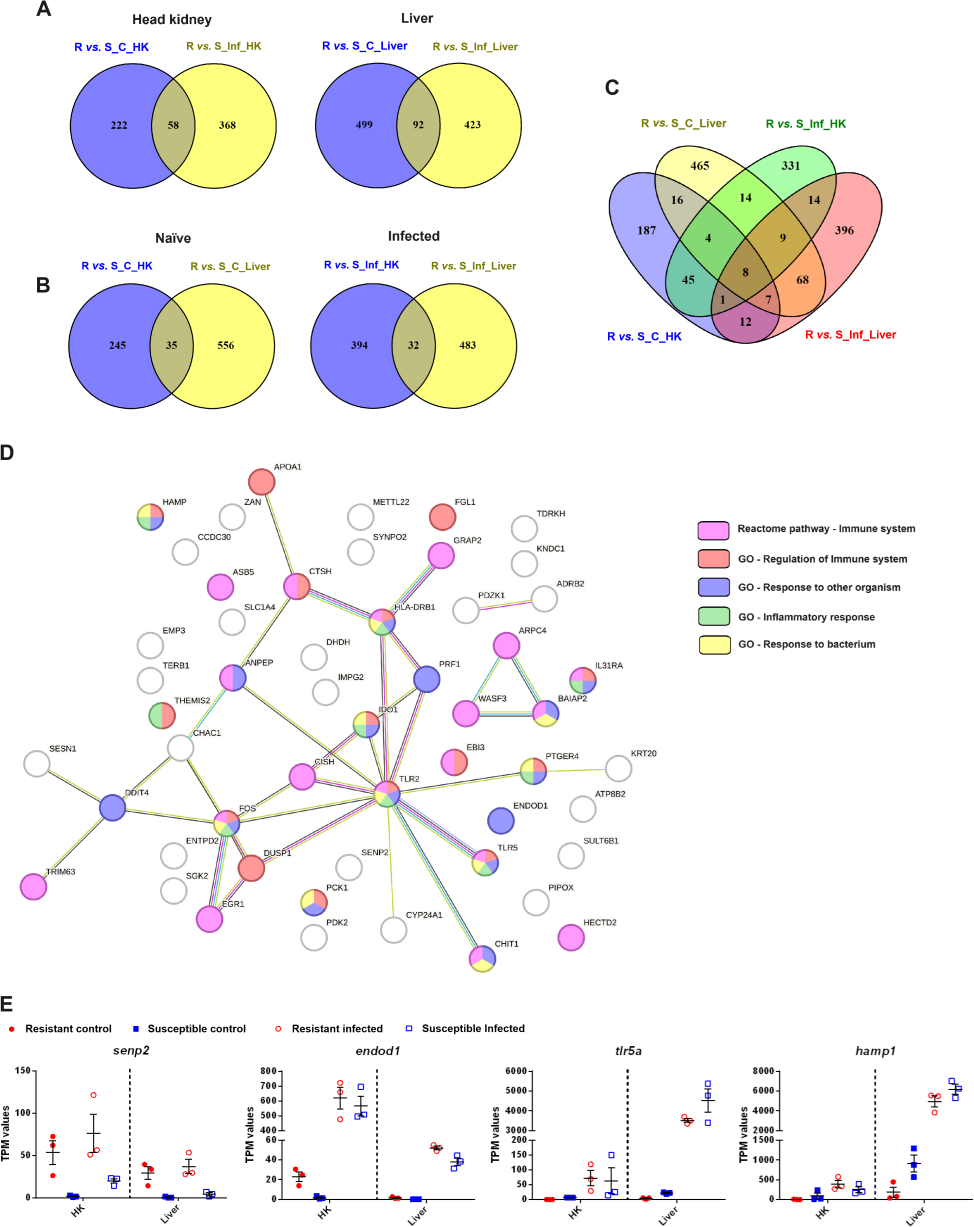
Certain DEGs between an *A*. *salmonicida*-resistant family and an *A*. *salmonicida*-susceptible family were differentially expressed both under naïve and infected conditions and in both target tissues (head kidney and liver). **(A)** Venn diagrams representing the DEGs between both families under naïve and infected conditions. A Venn diagram per tissue is shown. **(B)** Venn diagrams representing the common and exclusive DEGs between both families in the head kidney and liver. A Venn diagram per experimental condition (naïve or infected) is shown. **(C)** Venn diagram representing the DEGs between the resistant and susceptible turbot families under both experimental conditions and in both tissues. **(D)** STRING protein−protein interaction network representing the 59 proteins encoded by the genes commonly differentially expressed between the two families in both tissues (in the absence and/or presence of infection). The genes belonging to the significantly enriched Reactome pathways and GO biological process terms involved in the immune response are highlighted in different colors. **(E)** Representation of the expression (in TPM values) in the different experimental samples of four of these 59 genes commonly differentially expressed between turbot families in both tissues. The graphs represent the TPM values of the individual samples and the means ± SEMs.

Venn diagrams constructed with the DEGs between the resistant and susceptible families, both in the absence and presence of infection, revealed that while some genes were differentially expressed between the families under both conditions (58 in the head kidney and 92 in the liver), most of them were only differentially expressed under either naïve or infected conditions (**Figure 10A**). However, when the Venn diagrams were constructed by condition instead of by tissue, the number of shared DEGs was lower (35 DEGs for the naïve condition and 32 DEGs for the infected condition) (**Figure 10B**). However, these genes could be interesting marker genes for resistance, as they were differentially expressed in two functionally distant tissues. Information on the fold changes of these genes between families and how they responded to infection in both families is included in **Supplementary Table S4**. The inclusion of the four comparisons (R *vs*. S naïve and R *vs*. S infected in both tissues) revealed that only 8 genes were differentially expressed (**Figure 10C**). These genes corresponded to *senp2*, *hectd2*, Wiskott-Aldrich syndrome protein family member 3 (*wasf3*), protein very KIND (*kndc1*), tudor and KH domain-containing protein (*tdrkh*) and DNA damage-inducible transcript 4 protein (*ddit4*), which were expressed at higher levels in the resistant family in all cases, brain-specific angiogenesis inhibitor 1-associated protein 2 (*baiap2*), which was expressed at lower levels in the resistant family, and the uncharacterized protein LOC118315681, which was expressed at higher levels in the head kidney and lower levels in the liver than in the resistant family.

A protein−protein interaction network constructed with the 59 proteins encoded by the genes commonly differentially expressed between both families in both tissues revealed that they were involved mainly in the immune response (**Figure 10D**), and even included some proteins not classified in the immune reactome pathway or having immune GO terms, such as *senp2* and cytochrome P450 family 24 subfamily A member 1 (*cyp24a1*), that have relevant roles in the immune system.

Since those genes that were differentially expressed between two turbot families with different susceptibilities to *A. salmonicida* could also be a part of the characteristic response to the bacteria, information about how these genes were regulated after challenge in both families and tissues is shown in **Supplementary Table S4**. Some of these DEGs were significantly modulated *by A. salmonicida* in at least one of the families. This was the case for *senp2*, which was expressed at higher levels in the resistant family and significantly induced in both tissues after infection in the susceptible family, *endod1*, which was expressed at higher levels in the resistant family and significantly induced in both tissues after infection in both families, and *tlr5a* and *hamp1*, two genes strongly induced after bacterial challenge in both tissues from both families but showing lower basal expression in the resistant family (**Figure 10E**).

### 3.7 Integration of the transcriptome information and the major QTLs associated with *A. salmonicida* resistance in turbot

**Figure 11:**
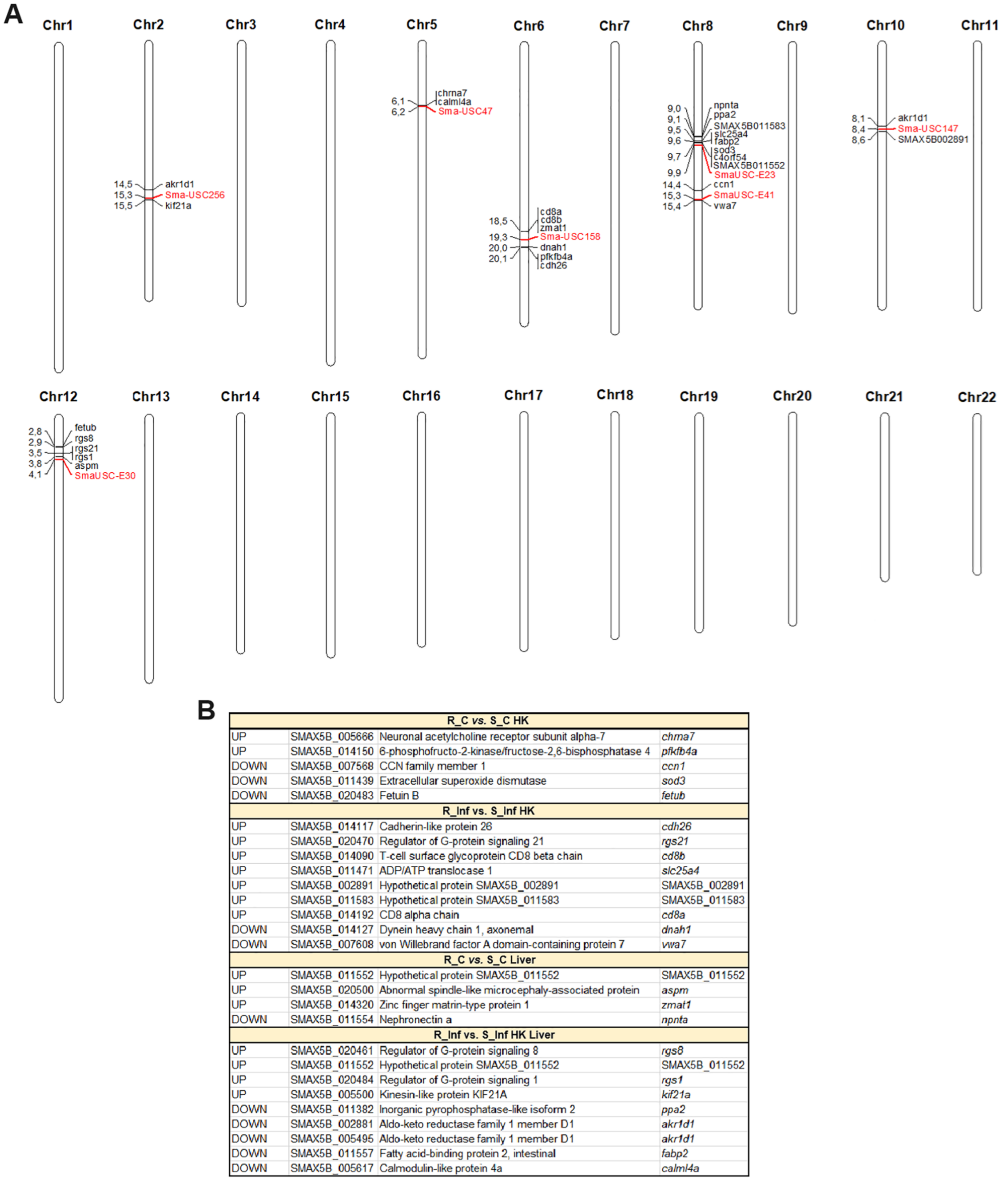
Differentially expressed genes between the resistant and susceptible turbot families (in at least one of the comparisons) located in *A*. *salmonicida* resistance QTLs. **(A)** Locations of the QTL-associated markers (in red color) and the surrounding DEGs (within a window of 2 Mbp upstream and downstream of the marker) on the turbot chromosomes. **(B)** List of the DEGs for the different comparisons of interest that are located in the vicinity of the QTL-associated markers. R, resistant; S, susceptible; C, control; Inf, infected.

The overlap of the DEGs between the resistant and susceptible families in some of the comparisons and QTL regions previously identified as associated with *A. salmonicida* resistance traits was explored (**Figure 11**). Genes that were differentially expressed were found in the vicinity of the 7 QTL-associated markers. Some of those DEGs play a role in the immune response, such as *cd8b* and *cd8a*, which are located in the QTL region of chromosome 6, different members of the regulator of the G protein signalling family (*rgs1*, *rgs8* and *rgs21*), which are located in the QTL region of chromosome 12, the antioxidant gene extracellular superoxide dismutase 3 (*sod3*), located in one of the QTLs found on chromosome 8, and the neurotransmitter receptor neuronal acetylcholine receptor subfamily alpha-7 (*chrna7*), which is positioned in the QTL region of chromosome 5, among others. Genes with a role in metabolism, such as fatty acid-binding protein 2 (*fabp2*), ADP/ATP translocase 1 (*slc25a4*) and 6-phosphofructo-2-kinase/fructose-2,6-biphosphatase 4 (*pfkfb4a*), were also associated with these QTLs. Interestingly, two genes with homology to aldo-keto reductase family 1 member D1 (*akr1d1*), encoding a protein primarily involved in bile acid synthesis, were associated with two different QTLs located on different chromosomes (chromosomes 2 and 10). Other genes overlapping with these QTLs were involved in cell adhesion (CCN family member 1 (*ccn1*), cadherin-like protein 26 (*cdh26*), von Willebrand factor A domain-containing protein 7 (*vwa7*), and nephronectin a (*npnta*)) or cytoskeletal organization (dynein heavy chain 1, axonemal (*dnah1*), abnormal spindle-like microcephaly associated protein (*aspm*), and kinesin-like protein KIF21a (*kif21a*)), among other functions. The complete repertoire of QTL overlapping genes is represented in **Figure 11**.

## 4 Discussion

For the first time, transcriptomic differences between two turbot families with divergent susceptibilities to *A. salmonicida* subsp. *salmonicida* were analyzed. Transcriptome profiles were examined both under naïve conditions and at 24 hpi, and the infection response of each family was also assessed. While a significant number of genes were commonly modulated following bacterial challenge in both families, an important subset of genes was uniquely modulated in both the resistant and susceptible families. This finding underscores the influence of genetic background on the pathogen response, which affects not only the magnitude of gene expression but also the specific nature of the modulation. Ours findings indicate that the liver under naïve conditions presented greater differences between families. High transcriptomic differences in liver samples were also observed in Japanese flounder families exhibiting varying susceptibilities to *E. tarda* (19), underscoring the importance of this tissue in influencing resistance to bacterial diseases.

In the head kidney, two of the most differentially regulated processes are the complement and coagulation pathways both in the absence and presence of infection. Interestingly, under naïve conditions, the resistant family presented lower expression of a multitude of key genes involved in this pathway, whereas the opposite pattern was observed in infected fish. Additionally, genes encoding certain AMPs, such as two genes annotated as perforin-1-like and *hamp1*, were expressed at lower levels in the resistant family. Although it may not seem so at first, a lower bacterial lysis rate during the early stages of infection in the resistant family could have a positive impact on survival. When bacteria lyse, they release their cellular contents, which can include toxins, enzymes, and other harmful molecules, into the surrounding environment (36). This release can exacerbate inflammation and tissue damage, contributing to the severity of bacterial infections (36). Therefore, while bacterial lysis is a natural process that occurs during infection and can aid in immune responses, excessive or uncontrolled lysis can be harmful to the host organism and lead to sepsis. In addition to its role as an antimicrobial peptide, hepcidin also controls bacterial growth by limiting iron availability, which is crucial for the metabolic processes of many bacteria (37). Serotransferrin and haptoglobin, which are also involved in iron sequestration and recycling, respectively (37), also exhibited reduced expression in the resistant family. Consequently, *A. salmonicida* could have more iron available for its growth in the resistant family. These observations could explain, at least in part, the absence of lower detection of *A. salmonicida* in the head kidney of the resistant family at 24 hpi. In contrast, the genes encoding other antimicrobial proteins, *nkl* and *pacap*, were found to be expressed at higher levels in head kidney samples from the resistant family. Nk-lysin, which is associated with resistance against VHSV in turbot, also presented a higher level of expression in head kidney samples from a resistant family under naïve conditions (26, 38). Surprisingly, Nkl was observed to be present in turbot red blood cells and localized within autophagosomes, suggesting additional roles linked to the autophagy process (38). Therefore, Nkl could contribute not only to the extracellular lysis of bacteria when released from the cytotoxic granules of NK cells and cytotoxic T lymphocytes but also to the intracellular degradation of bacteria. On the other hand, PACAP is a neuropeptide that, in recent years, has been shown to elicit antimicrobial activity in both mammals and fish (39, 40). However, this neuropeptide also plays a protective anti-inflammatory role during sepsis (41).

Additionally, under naïve conditions, genes involved in antigen recognition and presentation were expressed at higher levels in the resistant family. Higher expression of genes involved in antigen presentation was also observed in liver samples from *E. tarda*-resistant Japanese flounder at 24 hpi (23). Interestingly, whereas *tlr22*, encoding a teleost-specific toll-like receptor involved in the recognition of different viral and bacterial antigens (42), was highly expressed in the head kidney from the resistant family, *tlr2* and *tlr5a*, involved in the recognition of a variety of microbial structures (43) and bacterial flagellin (44), respectively, were expressed at lower levels in the resistant family. Although not differentially expressed among fry samples from three rainbow trout lines with different susceptibilities to *F. psychrophilum*, a gene annotated as *tlr5* was induced to higher levels after bacterial infection in the susceptible family than in the other families (24). In turbot, *tlr5a* was highly overexpressed after infection with *A. salmonicida* in both families, reaching similar expression values in infected fish.

In general, most of the remaining immune genes that were differentially expressed in head kidney samples between the two families under naïve conditions were significantly involved in regulating the inflammatory/tolerance response and the activation and function of B and T lymphocytes. This finding, combined with the lower expression of certain AMPs and TLRs and the higher expression of genes involved in antigen presentation–which, in turn, impacts T and B cell activation–in the resistant family, could suggest a more effective initiation of the adaptive immune response alongside a controlled inflammatory response. This predisposition to better initiation of the adaptive immune response, especially regarding T-cell activation, in the resistant family is also reflected in the higher expression of numerous genes related to the differentiation and activation of this cell type in the family resistant to *A. salmonicida* after infection. In the livers of Japanese flounder infected with *E. tarda*, several DEGs between a resistant line and a susceptible line were related to the T-cell receptor signalling pathway and presented increased expression in the resistant family (19). In terms of the inflammatory response, a greater induction of proinflammatory genes was observed after infection in rainbow trout fry from a family highly susceptible to *F. psychrophilum* than in those from two other families that presented greater resistance to this gram-negative bacterium (24) or in Atlantic salmon fries highly susceptible to IPNV compared to those from a family that presented good resistance to this virus (20). Consequently, it seems evident that a controlled inflammatory response provides more advantages in terms of survival than does an exacerbated response.

In the liver, a similar pattern of immune gene expression was observed. A lower expression of the genes encoding AMPs (perforin-1-like genes, *hamp1* and *leap2*) and *tlr2* and *tlr5a* was found, whereas other genes involved in antigen recognition and presentation were expressed at higher levels. Genes with a role in the regulation of the inflammatory/tolerance response and B and T lymphocyte activation and function also presented higher levels of expression in general in the resistant family. In addition to being the main metabolic organ, the liver also plays a fundamental role during the so-called acute phase of the immune response, a process that is rapidly activated following an infection and that is crucial for the regulation of the inflammatory response (45).The expression of the gene encoding the acute phase protein Gdf15, a hepatic pleiotropic cytokine considered a marker of sepsis severity both in mammals (46) and in fish (47), was higher in the livers of the susceptible family after infection. This observation could again suggest greater inflammatory damage in the susceptible family. Interestingly, the number of immune genes that were more highly expressed in the liver from the resistant family at 24 hpi was low. One of these genes was antibacterial L-amino acid oxidase (*il4i1*), which, through the production of hydrogen peroxide derived from the oxidation of L-amino acids, shows broad antibacterial activity (48). This antimicrobial gene was induced after bacterial challenge in both families both in head kidney (fold changes of 55.2 and 26.34 for the resistant and susceptible families, respectively, compared with the uninfected fish) and liver (fold changes of 1096.22 and 122.47 for the resistant and susceptible families, respectively, compared with the uninfected fish). Indeed, the *il4i1* gene presented the greatest level of induction after infection in the liver samples from the resistant family. Notably, among these DEGs in the liver at 24 hpi, there was an intriguing increase in the expression of genes involved in insulin and insulin-like growth factor (IGF) signalling in the resistant family after infection. Severe viral and bacterial infections are associated with an increase in systemic insulin resistance, which is mainly due to the downregulation of the insulin receptor and the alteration of the interaction between this insulin receptor and its adaptor molecules IRS1 and IRS2 mediated by the inflammatory response (49). Insulin resistance is therefore a common feature of sepsis, and insulin therapy improves sepsis outcomes (50). Bacteria can potentially benefit from insulin resistance due to the increased availability of glucose for their growth and replication and the impairment of the immune response (51). According to these results, the benefit for the resistant family seems evident, since increased expression of the genes involved in insulin signalling was observed. Since inflammation, insulin resistance and lipid metabolism are intimately interconnected (52), it is not surprising that some of the most highly enriched GO biological processes among the DEGs in the livers of the resistant and susceptible families after infection were related to lipid metabolism.

However, the main transcriptome differences observed in the liver samples between the two families are related to the activity of the cytoskeleton, which is pivotal for several immune processes, including phagocytosis (53), chemotaxis (54) and different steps of antigen presentation (55). Additionally, alterations in the cytoskeleton during bacterial infection play a crucial role in bolstering cell-intrinsic immunity. Specifically, these changes facilitate bacterial sensing, establish specialized subcellular compartments for distinct innate immune signalling pathways, provide architectural frameworks for pathogen sequestration, and orchestrate antibacterial mechanisms such as autophagy and host cell apoptosis (56). Overall, according to the gene expression analysis, liver cells from the resistant family presented increased cytoskeletal activity, including chemotaxis and phagocytosis, both under naïve and infected conditions. It has been previously shown that infection of turbot with *A. salmonicida* strongly inhibits the expression of a multitude of genes encoding cytoskeleton components in head kidney samples (27). Interestingly, in this work, strong inhibition after infection with *A. salmonicida* was observed in the livers of the susceptible family (**Supplementary File S4**) but not in those of the resistant family (**Supplementary File S3**). As occurs with several bacteria (57), the cytoskeleton and the extracellular matrix are manipulated by *A. salmonicida* during infection (58). This strategy aims to favor bacterial survival and spread (57, 59). We previously reported that pretreatment of turbot with the β-glucan zymosan A significantly mitigated the *A. salmonicida*-mediated inhibition of genes related to cytoskeletal dynamics in anterior kidney samples (28). Indeed, this mechanism could be related to the protection conferred by zymosan A against *A. salmonicida* (28). Related to the cytoskeleton, a multitude of genes involved in different steps of the cell cycle were differentially expressed in the liver between both families under both naïve and infected conditions, but especially under infected conditions. The expression results suggest that the liver cells from the resistant family had reduced mitotic activity (lower expression of genes favoring cycle progression and higher expression of two key genes involved in cell cycle arrest). Bacteria can arrest the host cell cycle through the secretion of virulence factors known as cyclomodulins to favor their own infective efficiency (60). Therefore, the lower division rate predicted for liver cells from the resistant family could be beneficial for bacteria on the basis of these premises. However, more investigations are needed to understand whether the lower mitotic activity of turbot liver cells could provide a beneficial effect against *A. salmonicida*. Additionally, motor proteins (mainly kinesins, myosins and troponins) were expressed at lower levels in the livers of the resistant family. However, whether this lower expression of motor proteins could have any effect on increased resistance in bacteria also remains to be elucidated.

A relatively low number of genes were commonly associated with resistance to *A. salmonicida* in both tissues. However, these genes could be of particular interest, as they may be good target candidates for testing in blood samples from fish showing varying susceptibilities to bacteria owing to their pleiotropic association with resistance. Unfortunately, blood samples were not taken in this study. However, the transcription level of the turbot *nkl* gene in blood cells, which was initially found to be highly expressed in head kidney samples from full-sibling families showing increased resistance to VHSV (26), was also positively correlated with increased resistance to VHSV (38). This approach could also serve as a nondestructive method for selective breeding, but the expression of those genes in blood and their relationship with resistance need to be validated. Most of these common DEGs were involved mainly in the immune response. For example, *senp2*, a gene that is known primarily for its role in SUMOylation and is involved in the control of the inflammatory response (61, 62), and *endod1*, which is involved both in DNA repair (63) and in the modulation of the cGAS−STING innate immunity pathway (64), were more highly expressed in the head kidney and liver of the resistant turbot family. Additionally, these genes were induced after bacterial challenge in both families and tissues, suggesting their relevance in the response to *A. salmonicida*. In this sense, the previous higher basal transcription of these genes could provide an advantage for survival during infection. In contrast, other genes, even when significantly and highly induced after challenge with bacteria in both families and tissues, were expressed at lower levels in the resistant family under naïve conditions. Examples include *tlr5a*, a flagellin-specific pathogen recognition receptor (44), and *hamp1*, an AMP also involved in iron homeostasis (65). In this case, as proposed above, a controlled inflammatory response during the early stages of infection could reduce inflammatory damage and, in turn, favor survival. Therefore, the measurement of these genes and other candidate genes in blood samples could serve as good nondestructive resistance markers that need to be validated.

Finally, we explored the overlap of the DEGs between both families and seven major QTLs associated with resistance to *A. salmonicida* subsp. *salmonicida* in turbot (15). The integration of both datasets revealed several DEGs located within QTL regions that could explain the association of these QTLs with resistance. While some of these genes were directly involved in the immune response, others were involved mainly in metabolism, cell adhesion, and cytoskeletal organization. However, further studies aimed at investigating the role of these genes in resistance to *A. salmonicida* could enhance our understanding of the molecular mechanisms underlying resistance to this pathogenic bacterium.

## 5 Conclusions

This study is the first to analyze the transcriptome profiles of two turbot families exhibiting different susceptibilities to a bacterial pathogen, specifically *A. salmonicida* subsp. *salmonicida*. RNA-Seq analyses revealed that even under naïve conditions, the resistant and susceptible families presented numerous DEGs in both the head kidney and liver. The results suggest that one of the mechanisms involved in resistance against the bacterium could be a controlled inflammatory response during the first hours after infection, increased antigen presentation and subsequent activation of the T-cell response, and increased control of cytoskeleton dynamics (involved in relevant immune processes such as phagocytosis and chemotaxis, among others). In conclusion, this work provides critical insights into the immune mechanisms underlying resistance to furunculosis in turbot and lays the foundation for future studies aimed at enhancing disease resistance in aquaculture. Those future studies could focus on the precise molecular pathways regulating these immune responses, with particular attention to how they can be modulated to improve disease resistance. Additionally, this information could be of great value in assisting with the development of selective breeding programs.

## Data Availability

The datasets presented in this study can be found in online repositories. The names of the repository/repositories and accession number(s) can be found in the article/**Supplementary Material**.

## Supplementary material

Supplementary Table 1 Primer pairs used in this work.

Supplementary Table 2 Length (cm) and weight (g) of a representative number (n=10) of turbot belonging to each full-sibling family before infection with *Aeromonas salmonicida* subsp. *salmonicida*. The mean and standard deviation (SD) were calculated for each family.

Supplementary Table 3 Summary of mRNA Illumina sequencing, trimming, and genome mapping.

Supplementary Table 4 Table of the shared DEGs between both turbot families in the head kidney and liver, both in the absence (R *vs*. S_C HK and R *vs*. S_C Liver) and presence of infection (R *vs*. S_Inf HK and R *vs*. S_Inf Liver). The names of the eight genes shared among the four sample groups are highlighted in grey. The green and red colors indicate higher or lower expression levels, respectively, of the DEGs in a certain comparison.

Supplementary Figure 1 Validation of the RNA-Seq results by qPCR. Three genes implicated in the immune response and exhibiting differential expression between both full-sibling turbot families in both the head kidney and liver were selected.

Supplementary Figure 2 Heatmap representing cytoskeleton-related DEGs in liver samples from resistant and susceptible turbot families at 24 hpi with *A. salmonicida*.

Supplementary Figure 3 KEGG pathway summarizing the motor proteins and the differential expression of their encoding genes in liver samples from resistant and susceptible turbot families. Green indicates higher expression, and red indicates lower expression in the resistant family compared to that in the susceptible family. The dashed line represents the DEGs between both families under naïve conditions, whereas the solid line represents the DEGs between both families at 24 hpi with *A. salmonicida*.

Supplementary Figure 4 Heatmap representing DEGs in liver samples from resistant and susceptible turbot families at 24 hpi with *A. salmonicida* involved in lipid metabolism.

Supplementary File S1 Differentially expressed genes (DEGs) between the resistant and susceptible families under naïve and *A. salmonicida*-infected conditions in head kidney samples.

Supplementary File S2 Differentially expressed genes (DEGs) between the resistant and susceptible families under naïve and *A. salmonicida*-infected conditions in liver samples.

Supplementary File S3 Differentially expressed genes (DEGs) in the resistant family at 24 hpi with *A. salmonicida* in the head kidney and liver.

Supplementary File S4 Differentially expressed genes (DEGs) in the susceptible family at 24 hpi with *A. salmonicida* in the head kidney and liver.
