# Supplementary material for: Unravelling turbot (Scophthalmus maximus) resistance to Aeromonas salmonicida: transcriptomic insights from two full-sibling families with divergent susceptibility
Source: Front Immunol. 2024 Dec 6;15:1522666. doi: 10.3389/fimmu.2024.1522666 (PMC11659141; doi:10.3389/fimmu.2024.1522666)

Supplementary Material

1 Supplementary Tables

Supplementary Table S1. Primer pairs used in this work.

Gene name	Forward	Reverse
A. salmonicida specific sequence	CGTAATCTGAATTGTTCTTTTCCG	ATTGCTTATCGAGGCAGCCAAC
senp2	AGGGAGGTTGAGAGTGAGGT	TGCCGATTGTCACAGACCAG
hectd2	GGTCAGTAAAGTGTCCCGCA	CCGCTTCCTCGTCAACTCAT
tlr5a	TGGCAGAAGTGTGGGAATCC	CAGTTTGAGGTGGGCGATCT
eef1a	GGAGGCCAGCTCAAAGATGG	ACAGTTCCAATACCGCCGATTT

Supplementary Table S2. Length (cm) and weight (g) of a representative number (n=10) of turbot belonging to each full-sibling family before infection with *Aeromonas salmonicida* subsp. *salmonicida*. The mean and standard deviation (SD) were calculated for each family.

	22035		22038		22037		22039		22040	
	**Length (cm)**	**Weight (g)**	**Length (cm)**	**Weight (g)**	**Length (cm)**	**Weight (g)**	**Length (cm)**	**Weight (g)**	**Length (cm)**	**Weight (g)**
**1**	8.4	9.3	8.9	11.3	9.1	12.9	8.1	9.5	8.9	11.2
**2**	9	11.2	9	11.4	8.5	10	8.3	9.9	8.5	9.9
**3**	8.8	10.4	9	12.1	8.4	9.9	8.9	12.2	9	11.4
**4**	8.9	11.1	8.7	10.7	8.8	11.5	9.2	14.4	8.4	10
**5**	8.7	11	8.5	9.9	8.7	11.2	8.3	9.6	8.1	8.9
**6**	8.7	10.6	8.9	11.7	8.6	10.7	8.3	10.3	8.8	10.3
**7**	9.2	12.6	8.9	10	8.1	9.8	9.2	14.6	9	13
**8**	9	12.1	8.7	10.7	8.5	10.6	8.1	9.1	8.8	11.3
**9**	8.5	9.9	8.8	10.2	8.8	11.2	8.8	11.4	8.4	9.9
**10**	9.4	14	9.5	14	8.9	11.5	8.6	10.7	8.6	10.6
**MEAN**	8.86	11.22	8.89	11.2	8.64	10.93	8.58	11.17	8.65	10.65
**SD**	0.31	1.37	0.26	1.23	0.28	0.95	0.42	1.98	0.30	1.13

Supplementary Table S3. Summary of mRNA Illumina sequencing, trimming, and genome mapping.

Sample	Raw reads	Reads after trimming	Mapped to genome (%)
**R_C HK1**	24,502,854	24,502,841	90.62
**R_C HK2**	29,838,368	29,838,353	87.51
**R_C HK3**	28,399,614	28,399,606	84.46
**S_C HK 1**	25,465,266	25,465,263	83.67
**S_C HK 2**	28,389,826	28,389,816	86.51
**S_C HK 3**	26,571,676	26,571,673	83.12
**R_Bac HK1**	25,265,596	25,265,590	82.97
**R_Bac HK2**	21,070,732	21,070,726	77.74
**R_Bac HK3**	26,142,938	26,142,933	82.35
**S_Bac_HK 1**	26,623,780	26,623,772	88.72
**S_Bac_HK 2**	29,782,192	29,782,189	84.59
**S_Bac_HK 3**	27,278,020	27,278,002	79.61
**R_C Liver 1**	26,514,520	26,514,516	80.17
**R_C Liver 2**	28,230,146	28,230,143	90.01
**R_C Liver 3**	33,178,340	33,178,193	88.88
**S_C Liver 1**	25,835,386	25,835,386	82.73
**S_C Liver 2**	24,799,386	24,799,376	55.96
**S_C Liver 3**	33,235,848	33,235,839	57.15
**R_Bac Liver 1**	29,577,968	29,577,955	79.53
**R_Bac Liver 2**	25,614,008	25,613,989	80.26
**R_Bac Liver 3**	21,994,470	21,994,439	77.3
**S_Bac Liver 1**	21,363,000	21,363,000	85.88
**S_Bac Liver 2**	26,972,116	26,972,066	86.33
**S_Bac Liver 3**	33,300,676	33,300,647	80.44

Supplementary Table S4. Table of the shared DEGs between both turbot families in the head kidney and liver, both in the absence (R vs. S_C HK and R vs. S_C Liver) and presence of infection (R vs. S_Inf HK and R vs. S_Inf Liver). The names of the eight genes shared among the four sample groups are highlighted in grey. The green and red colours indicate higher or lower expression levels, respectively, of the DEGs in a certain comparison.

		Head kidney				Liver			
Gene ID	Annotation	R_C vs. S_C_HK	R_Inf vs. S_Inf_HK	R_Inf vs. R_C_HK	S_Inf vs. S_C_HK	R_C vs. S_C_Liver	R_Inf vs. S_Inf_Liver	R_Inf vs. R_C_Liver	S_Inf vs. S_C_Liver
SMAX5B_020675	Sentrin-specific protease 2	28.98	3.89	----	12.10	51.56	9.18	----	7.15
SMAX5B_002496	Endonuclease domain-containing 1 protein	10.82	----	32.49	319.33	10.96	----	43.46	343.73
SMAX5B_010475	Prostaglandin E receptor 4 (subtype EP4) a	6.85	----	----	8.84	8.63	----	-7.88	----
SMAX5B_017746	E3 ubiquitin-protein ligase HECTD2	6.24	4.29	----	----	9.30	7.83	----	----
SMAX5B_021582	Wiskott-Aldrich syndrome protein family member 3	4.58	5.57	----	----	5.19	7.27	----	----
SMAX5B_003838	uncharacterized protein LOC118315681	3.99	3.37	----	----	-6.40	-7.13	----	----
SMAX5B_019080	Ankyrin repeat and SOCS box protein 5	3.92	----	----	----	3.07	4.07	----	----
SMAX5B_002370	Epithelial membrane protein 3	3.74	----	-2.83	----	2.88	----	-3.10	----
SMAX5B_008705	Protein very KIND	3.64	4.72	----	----	3.88	4.63	----	-2.08
SMAX5B_016702	Tudor and KH domain-containing protein	2.83	2.60	----	----	4.58	3.14	----	----
SMAX5B_012124	B-lymphocyte antigen CD20	2.71	2.35	----	----	7.72	----	----	----
SMAX5B_001167	wu:fc46h12 precursor	2.51	----	----	----	11.08	----	----	----
SMAX5B_018780	Ectonucleoside triphosphate diphosphohydrolase 2	2.41	----	----	2.03	4.07	----	----	----
SMAX5B_009907	Pro-cathepsin H	2.33	----	----	----	2.63	----	----	2.91
SMAX5B_005297	Hypothetical protein SMAX5B_005297	2.30	3.09	----	----	3.57	----	----	----
SMAX5B_015986	GRB2-related adapter protein 2a	2.20	----	----	----	5.87	----	----	----
SMAX5B_012136	Phospholipid-transporting ATPase ID (ATPase class I type 8B member 2)	2.18	2.25	----	----	2.89	----	-2.99	----
SMAX5B_012077	Protein THEMIS2	2.14	----	----	----	5.85	----	----	----
SMAX5B_017367	DNA damage-inducible transcript 4 protein	2.13	2.35	----	----	10.72	5.89	-7.57	-4.16
SMAX5B_022001	Interleukin-27 subunit beta	2.12	----	15.85	27.54	4.70	----	-2.95	----
SMAX5B_001378	Methyltransferase-like protein 22	2.04	----	----	----	2.43	2.80	----	----
SMAX5B_010559	MHC class II beta antigen	2.04	----	----	----	2.78	----	-2.37	----
SMAX5B_003284	Serine/threonine-protein kinase Sgk2	-2.05	----	----	----	-2.09	-3.67	-9.60	-5.47
SMAX5B_008589	Neutral amino acid transporter A	-2.24	----	----	----	-2.36	----	3.49	----
SMAX5B_004468	Toll-like receptor 2	-2.28	----	----	----	-2.15	----	2.38	----
SMAX5B_008453	Peroxisomal sarcosine oxidase	-2.38	----	-2.38	-4.12	2.88	----	-3.87	----
SMAX5B_003720	Hepcidin 1 precursor	-3.69	----	78.70	13.92	-15.04	----	77.22	6.37
SMAX5B_010082	Telomere repeats-binding bouquet formation protein 1	-3.73	----	----	----	-8.09	----	----	-6.07
SMAX5B_020616	Brain-specific angiogenesis inhibitor 1-associated protein 2	-4.54	-3.68	----	----	-2.50	-2.62	----	----
SMAX5B_017556	Zonadhesin-like isoform 2	-5.90	----	----	-8.69	-32.62	-21.94	----	----
SMAX5B_000015	Toll-like receptor 5a	-7.27	----	273.73	16.03	-3.24	----	688.57	269.25
SMAX5B_011010	Indoleamine 2,3-dioxygenase 1	-8.60	-2.62	2.85	----	2.12	----	----	----
SMAX5B_018752	Perforin-1	-11.91	----	----	-8.77	-2.43	-2.51	----	----
SMAX5B_000329	Fibrinogen-like protein 1	-12.26	----	----	-17.69	-2.25	-8.68	-16.13	-4.19
SMAX5B_006098	Regakine-1	-14.89	----	----	-107.31	-2.64	-2.13	----	----
SMAX5B_005208	Cytochrome P450 Family 24 Subfamily A Member 1 (Cyp24a1l)	----	33.04	----	-55.88	2.04	2.15	----	----
SMAX5B_011762	Apolipoprotein A-I	----	6.80	----	-52.34	----	-2.20	-4.49	----
SMAX5B_016443	Cytosolic phosphoenolpyruvate carboxykinase	----	5.22	-4.34	-30.68	----	3.04	-4.21	-9.71
SMAX5B_017375	Synaptopodin 2	----	3.88	----	----	-23.26	-19.98	----	-3.43
SMAX5B_019618	Sestrin-1	----	2.91	2.28	----	----	2.06	----	----
SMAX5B_000349	Glutathione-specific gamma-glutamylcyclotransferase 1	----	2.86	----	----	2.51	4.85	-2.60	-5.02
SMAX5B_002527	Hypothetical protein SMAX5B_002527	----	2.85	----	-2.54	----	3.80	----	----
SMAX5B_013103	Trans-1,2-dihydrobenzene-1,2-diol dehydrogenase	----	2.53	----	----	5.74	9.95	----	-2.86
SMAX5B_012241	Early growth response protein 1	----	2.52	5.67	----	-5.75	-3.59	----	-3.02
SMAX5B_013971	Interleukin-31 receptor subunit alpha	----	2.51	----	----	4.26	2.59	----	----
SMAX5B_002325	Cytokine-inducible SH2-containing protein	----	2.45	----	----	----	3.87	-4.39	-9.20
SMAX5B_008861	Early growth response protein 1	----	2.44	3.45	----	-8.38	-3.52	----	-2.75
SMAX5B_017751	Sulfotransferase 6B1	----	2.39	----	-2.21	----	-3.02	----	----
SMAX5B_002139	E3 ubiquitin-protein ligase TRIM63a	----	2.32	----	----	2.79	3.70	-4.11	-5.46
SMAX5B_013017	Pyruvate dehydrogenase (acetyl-transferring) kinase isozyme 2, mitochor	----	2.20	2.53	----	2.84	3.51	-2.55	-3.15
SMAX5B_002836	Proto-oncogene protein c-Fos	----	2.18	4.95	2.03	----	2.91	----	----
SMAX5B_015407	Aminopeptidase N	2.94	2.15	----	2.56	----	-1852.92	----	2025.53
SMAX5B_002357	Actin-related protein 2/3 complex subunit 4	----	2.08	----	-2.15	----	2.67	----	----
SMAX5B_002106	Interphotoreceptor matrix proteoglycan 2	----	2.06	5.85	3.16	----	-3.57	----	5.15
SMAX5B_018920	Dual specificity protein phosphatase	----	2.01	3.93	----	----	2.32	5.94	5.45
SMAX5B_021331	Na(+)/H(+) exchange regulatory cofactor NHE-RF3	----	-2.00	----	----	----	-19.15	----	----
SMAX5B_014809	Coiled-coil domain-containing protein 30	----	-2.09	----	----	----	-4.97	-4.72	----
SMAX5B_015485	Chitin synthase 1	----	-2.33	----	5.54	----	-3.48	6.06	25.20
SMAX5B_006791	Beta-2 adrenergic receptor	----	-5.14	----	----	----	-8.63	----	----

2 Supplementary Figures

Supplementary Figure S1. Validation of the RNA-Seq results by qPCR. Three genes implicated in the immune response and exhibiting differential expression between both full-sibling turbot families in both the head kidney and liver were selected.

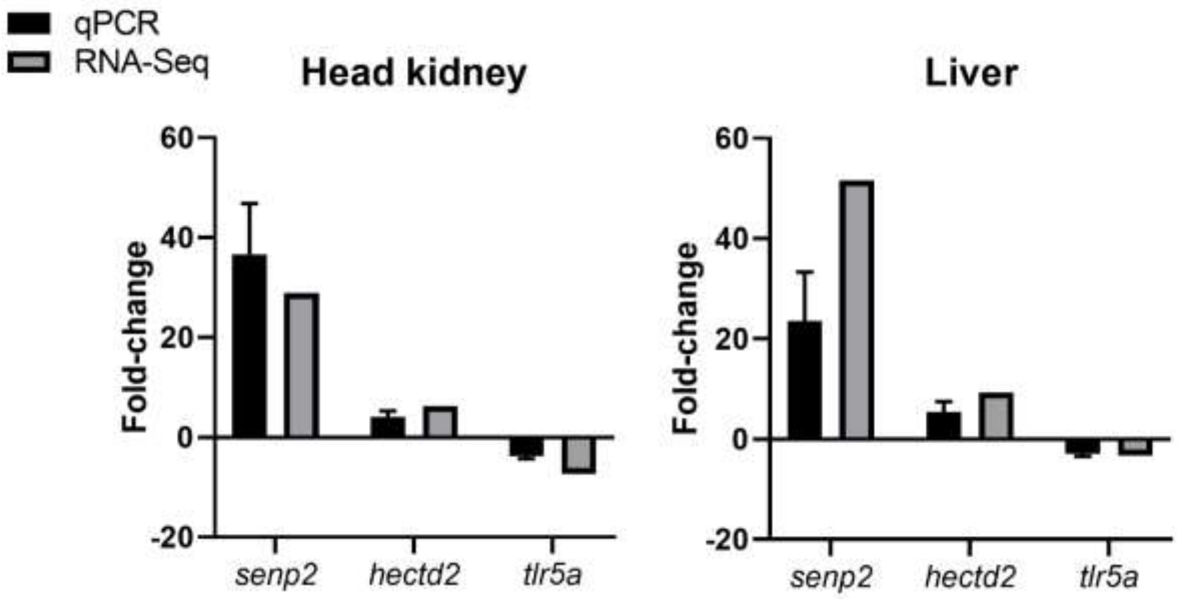

Supplementary Figure S2. Heatmap representing cytoskeleton-related DEGs in liver samples from resistant and susceptible turbot families at 24 hpi with *A. salmonicida*. The scale bar represents the Z-score of the TPM values for each row.

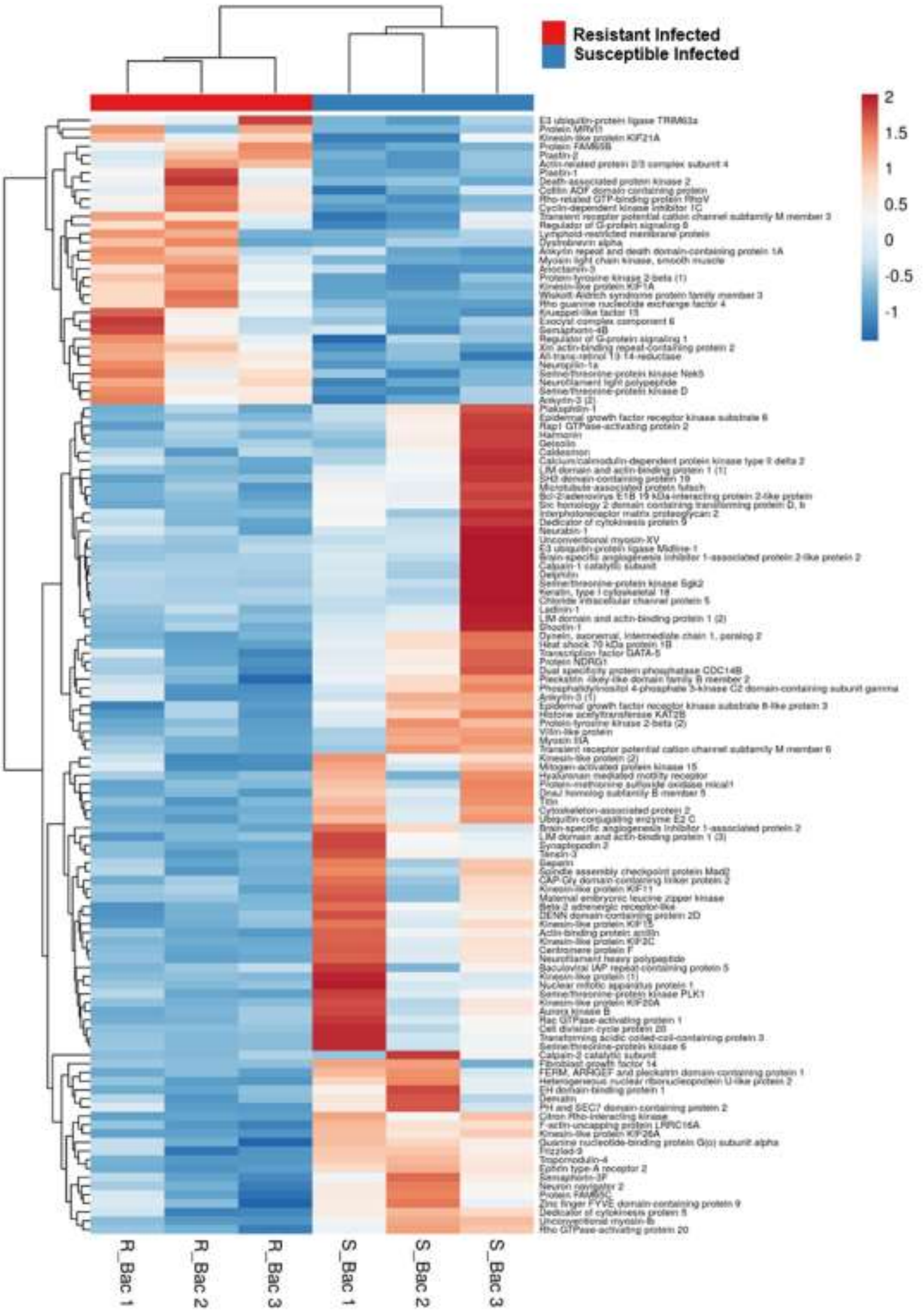

Supplementary Figure S3. KEGG pathway summarizing the motor proteins and the differential expression of their encoding genes in liver samples from resistant and susceptible turbot families. Green indicates higher expression, and red indicates lower expression in the resistant family compared to that in the susceptible family. The dashed line represents the DEGs between both families under naïve conditions, whereas the solid line represents the DEGs between both families at 24 hpi with *A. salmonicida*.

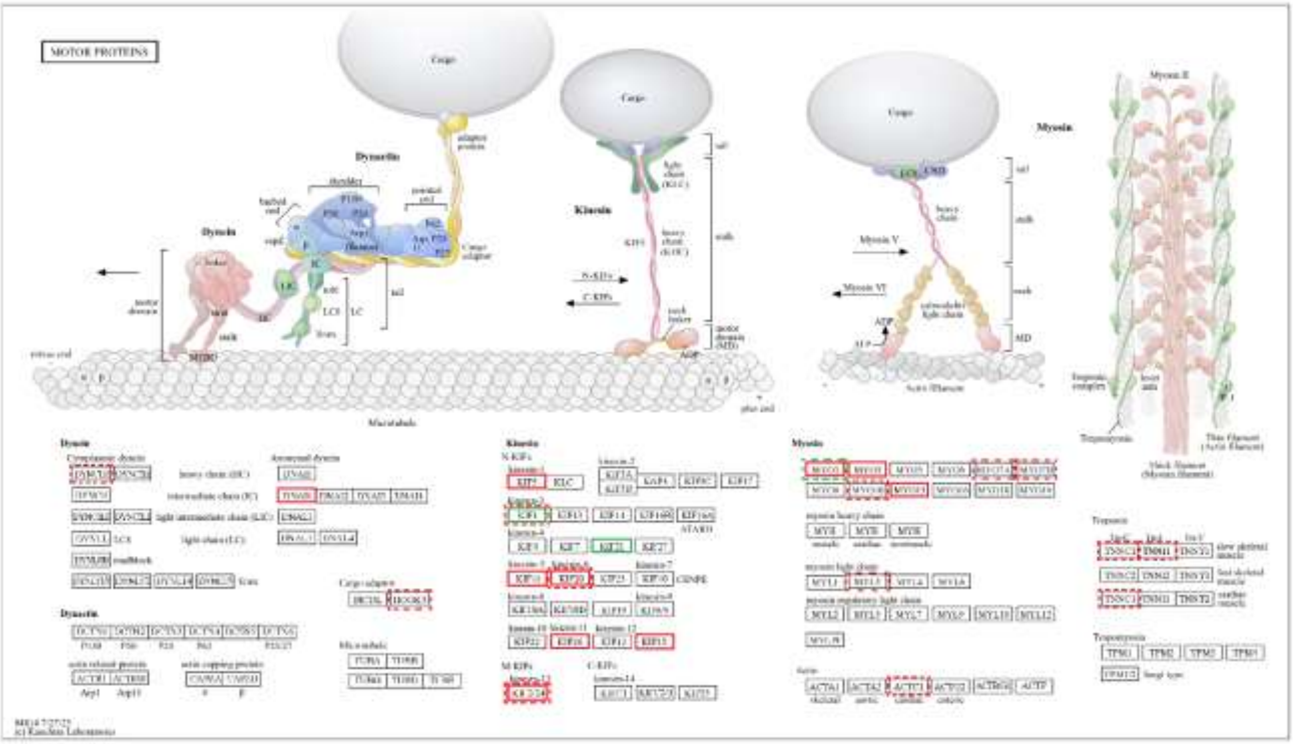

Supplementary Figure S4. Heatmap representing DEGs in liver samples from resistant and susceptible turbot families at 24 hpi with *A. salmonicida* involved in lipid metabolism. The scale bar represents the Z-score of the TPM values for each row.

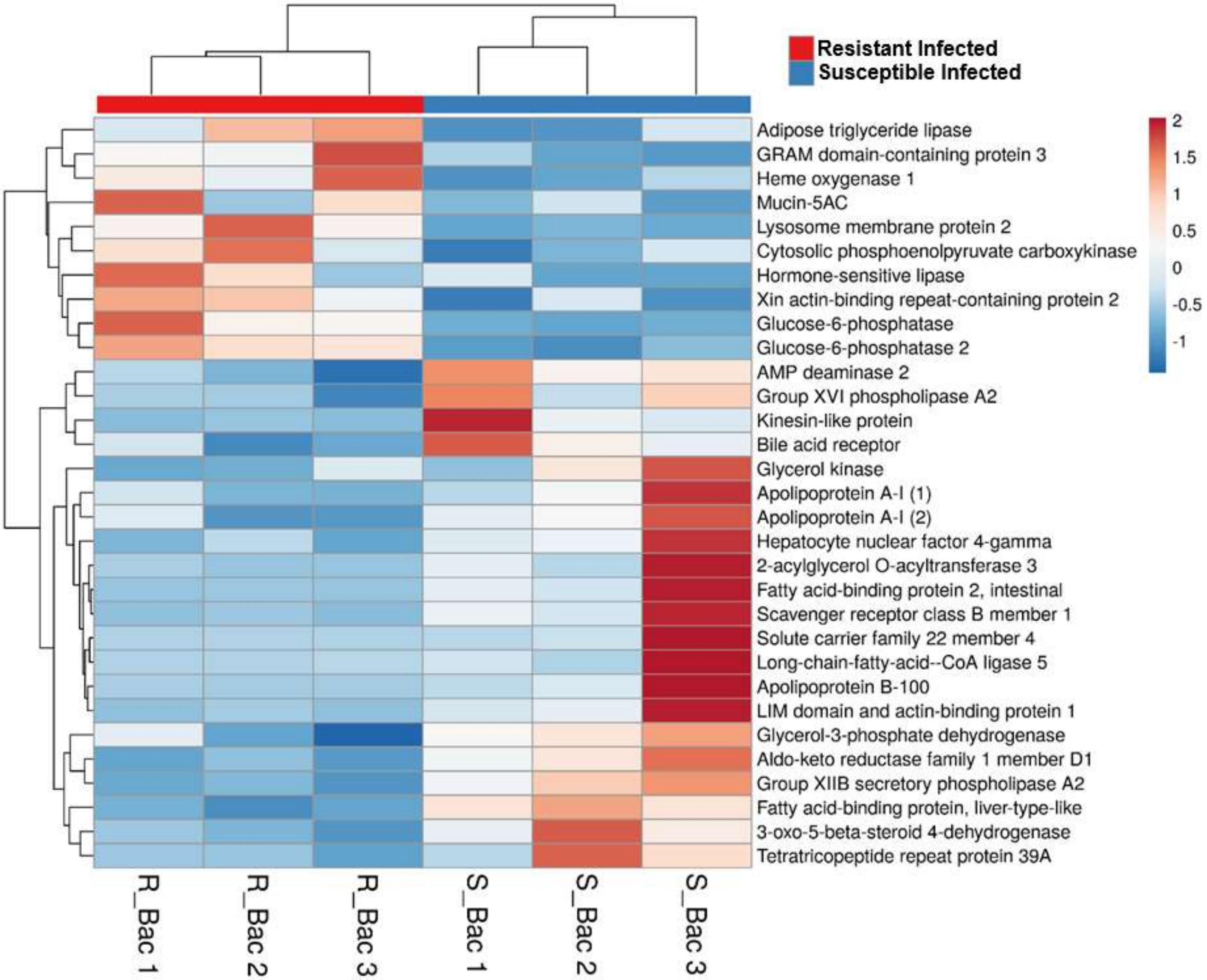

Supplement: Supplementary Table 1 — Primer pairs used in this work. [file DataSheet1.pdf]
